# Differential analysis between somatic mutation and germline variation profiles reveals cancer-related genes

**DOI:** 10.1186/s13073-017-0465-6

**Published:** 2017-08-25

**Authors:** Pawel F. Przytycki, Mona Singh

**Affiliations:** 1 Department of Computer Science, Princeton University, Princeton, NJ 08544 USA; 2 Lewis-Sigler Institute for Integrative Genomics, Princeton University, Princeton, NJ 08544 USA

**Keywords:** Cancer, Whole-exome sequencing, Somatic mutations, Germline variation, Cancer driver genes

## Abstract

A major aim of cancer genomics is to pinpoint which somatically mutated genes are involved in tumor initiation and progression. We introduce a new framework for uncovering cancer genes, differential mutation analysis, which compares the mutational profiles of genes across cancer genomes with their natural germline variation across healthy individuals. We present DiffMut, a fast and simple approach for differential mutational analysis, and demonstrate that it is more effective in discovering cancer genes than considerably more sophisticated approaches. We conclude that germline variation across healthy human genomes provides a powerful means for characterizing somatic mutation frequency and identifying cancer driver genes. DiffMut is available at https://github.com/Singh-Lab/Differential-Mutation-Analysis.

**Electronic supplementary material:**

The online version of this article (doi:10.1186/s13073-017-0465-6) contains supplementary material, which is available to authorized users.

## Background

Large-scale cancer genome sequencing consortia, such as TCGA [1] and ICGC [2], have provided a huge influx of somatic mutation data across large cohorts of patients. Understanding how these observed genetic alterations give rise to specific cancer phenotypes represents a major aim of cancer genomics [3]. Initial analyses of cancer genomes have revealed that numerous somatic mutations are usually observed within each individual and yet only a subset of them is thought to play a role in tumor initiation or progression [4]. Further, such analyses have shown that somatic mutations in cancer are highly heterogeneous, with each individual presenting a distinct set of mutations across many genes [3, 4]. As a result, computational methods are necessary for analyzing cancer genomics datasets in order to uncover which of the many observed altered genes are functionally important in cancers [5].

Perhaps the most commonly applied approach to identify cancer-related genes is to analyze a cohort of individuals and find the genes in which somatic mutations frequently occur [6, 7]. However, gene-specific characteristics, such as length, replication timing, and expression, all play a role in any given gene’s propensity for acquiring mutations [4, 5, 7, 8]. Thus, a gene’s frequency of mutation is typically compared to a background mutation rate, computed across either the entire gene or a specific genomic region, that represents how frequently we would expect that gene to be mutated by chance alone; only genes with mutation rates significantly higher than background mutation rates are predicted to be relevant for cancer [8–12]. Background mutation rates have been estimated based upon a variety of data, including silent mutation frequency [11, 12], mutational frequencies per nucleotide contexts (e.g. CG dinucleotides) [9], and known gene-specific characteristics [8, 10], as well as combinations of these features as inferred using machine learning techniques [13]. A high background mutation rate in a gene is indicative of that gene’s propensity to accumulate mutations, thereby suggesting that mutations within it are more likely to be neutral [11].

Here we introduce a new framework, differential mutation analysis, that uncovers cancer genes by comparing the mutational profiles of genes across cancer genomes with their natural germline variation profiles across healthy individuals. We hypothesize that if a gene is less constrained with respect to variation across the healthy population, it may also be able to tolerate a greater amount of somatic mutation without experiencing a drastic detrimental functional change. Our rationale is that the propensity of a gene to acquire neutral mutations is likely subject to many of the same gene specific characteristics (e.g. length) regardless of whether these mutations occur in germline cells or somatic cells [6, 14]. Furthermore, genomic breakpoints tend to be shared across genomic samples leading to instability and mutations in the same regions in both somatic and germline cells [15]. Thus, we propose that just as differential gene expression analysis in cancer studies identifies genes that are differentially expressed between cancer samples and normal samples, so differential mutation analysis can reveal genes that are differentially mutated between cancer genomes and the genomes of healthy individuals. While genes that are found to be differentially expressed are thought to reflect functional differences in regulation [16], we propose that genes that are differentially mutated are candidate cancer “driver” genes.

We present a fast and simple method for differential mutational analysis. Our approach leverages large-scale human variation data from the 1000 Genomes project [17] and identifies genes whose mutational profiles across cancer genomes are enriched compared to their relative variability across healthy populations. Previously, natural variation data have been used to interpret mutations found in the genomes of individuals with a disease of interest [12, 18–20]. For example, mutations that fall in highly polymorphic sites are frequently assumed not to play a significant role in disease [12, 18, 19]. Furthermore, genic regions with a high ratio of rare variants to common ones have been found to be more intolerant to functional variation and thus changes within them are more likely to be responsible for inherited diseases [20]. Somatic mutations that fall into such regions can also have a large functional impact [18, 19]. Moreover, per-gene rare variant frequency has been used to prioritize cancer genes and distinguish tumor samples from normal samples [21]. In contrast to these earlier approaches that consider allelic frequencies at individual sites to help elucidate the impact of mutations, our work introduces the idea of comparing the variability of a gene across a healthy population with its mutational profile across a cancer cohort in order to determine whether it is likely to be relevant for cancer.

Our method for identifying genes differentially mutated in cancer does not rely on any parameter fitting or machine learning and obviates the need to integrate the large amounts of external covariate data that many other methods rely on [7]. Our method runs in minutes and outperforms considerably more sophisticated and time-consuming approaches for uncovering cancer genes. We therefore posit that germline variation information can serve as a robust background for characterizing somatic mutations revealed by cancer genome sequencing studies and that differential mutation analysis is an intuitive yet highly efficacious framework for discovering cancer driver genes.

## Methods

### Method overview

**Fig. 1:**
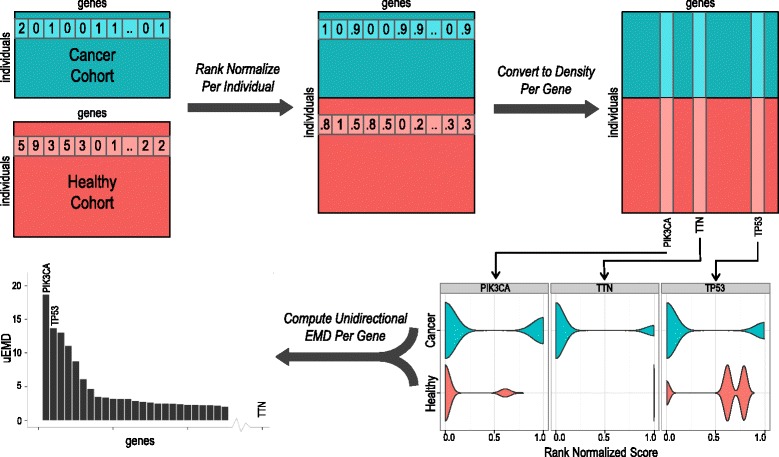
Overview of the differential mutation framework. Our method evaluates each gene for differential mutation when comparing cancer and healthy cohorts. For a cancer type of interest, we first count, for each individual, the number of somatic mutations found in each gene. Similarly, we use the 1000 Genomes sequencing data to count, for each individual, how many variants appear in each gene (*top left*). For each individual, we rank normalize the genes so that each gene has a score between 0 and 1 that reflects the relative number of mutations or variations that fall within it, compared to other genes within that individual (*top middle*). Next, for each gene, we aggregate its mutation and variation scores across healthy and cancer cohorts separately, resulting in a set of normalized variation scores as well as a set of normalized mutation scores (*top right*). We use each of these sets to build a histogram estimating the density of mutation or variant normalized scores. Shown here are the smoothed densities for the three most mutated genes in breast cancer (*bottom right*). Finally, in order to uncover whether a gene has a mutational profile that is very different between natural and cancer cohorts, we compute the difference between the two distributions using a modification of the classical Earth Mover’s Distance, which we refer to as a unidirectional Earth Mover’s Difference (uEMD). Genes with large differences between the two distributions are predicted as cancer genes (*bottom left*). See “Methods” for details

We have developed a method, DiffMut, that evaluates each gene for differential mutation when comparing cancer and healthy cohorts. Our approach is entirely based on somatic mutations and germline variation, without any additional parameters (Fig. 1). Briefly, for a cancer type of interest, we first count, for each individual, the number of non-silent single nucleotide mutations found in the exons of each gene. Similarly, we use the 1000 Genomes sequencing data to count, for each individual, how many variants appear in each gene. We define a variant as any nucleotide that differs from the most common one across the healthy cohort. For each individual, we then rank normalize the mutation or variant counts across genes so that each gene is assigned a score between 0 and 1 that reflects the relative number of mutations or variants that fall within it. Next, for each gene, we aggregate its mutation and variation scores across healthy and cancer cohorts separately, resulting in a set of normalized variation scores as well as a set of normalized mutation scores. We use these sets to build a pair of histograms estimating the density of mutation and variant normalized scores. The first represents the gene’s ranks among all genes with respect to somatic mutation across a cancer genome cohort; the other represents its ranks with respect to germline variation across a healthy cohort. In order to uncover whether a gene has a mutational profile that is more extreme for cancer than healthy cohorts, we compute the difference between the two distributions using a modification of the classic Earth Mover’s Distance [22], which we refer to as a unidirectional Earth Mover’s Difference (uEMD). A key advantage of an EMD-based score is that it measures the cost of transforming one distribution into another by considering the shapes of the two distributions in addition to the differences between the constituent values. Genes with higher uEMD scores have normalized cancer mutation scores that tend to be larger than their normalized variation scores. Thus, we rank all genes by their uEMD scores, considering higher ranking genes to be more likely to be functionally related to a given cancer type, and compute a supporting empirical *q*-value at each uEMD score [23].

### Processing cancer exome mutations

We downloaded all level 3 cancer somatic mutation data from The Cancer Genome Atlas (TCGA) [1] that was available as of October 1, 2014. This consisted of 75 Mutation Annotation Format (MAF) files across 24 cancer types. We then mapped point mutations based on their provided location in the human reference genome to all known human proteins in NCBI’s annotation release 104 whose amino acid sequences matched nucleotide sequences from the human reference genome build 37 patch 10 (GRCh37.p10) [24]. Mutations were classified as missense if they changed the encoded amino acid, nonsense if they changed an amino acid into a stop codon, and silent if they had no effect on the protein sequence. For each gene, we selected only the longest known isoform, which left us with 19,460 protein isoforms that uniquely mapped to genes. In cases where the MAF file was annotated to an earlier release of the human reference genome, we used the liftOver tool [25] to convert genomic locations to build 37. For each of the 24 cancer types, we selected the MAF file with the most mapped non-silent mutations (with the exception of those files processed by Canada’s Michael Smith Genome Sciences Centre which excluded nonsense mutations) in order to have the largest number of mutations without mixing mutations from different processing pipelines (see Additional file 1: Section A for mutation counts for each cancer type).

### Processing natural human variants

We downloaded all phase 3 whole-genome variant calls from the 1000 Genomes Project (released May 2, 2013) [17] and mapped them uniquely to the longest isoform for each gene as described above. This resulted in 960,408 variant sites over 2504 healthy individuals, of which 578,002 contained missense variants, 11,543 contained nonsense variants, and 370,974 contained silent variants (note that a single variant site can yield missense, silent, or nonsense variations in different individuals). For each variant site, each individual is given a score of 0, 1, or 2 depending upon whether the variant is absent, heterozygous, or homozygous relative to the most commonly observed allele in the population. Variants in the Y chromosome were excluded and variants in male X chromosomes were always marked as homozygous.

### Rank normalizing mutations and variation counts per individual

For each individual with cancer, we counted the number of mutations that were found in each gene in their cancer genome. Similarly, for each individual included in the 1000 Genomes Project, we counted the sum of variant scores for each gene, as described above. Next, for each individual, we rank normalized their mutation or variation counts across all genes. To do so, each gene was first assigned a rank equal to the number of genes it had a greater count than. All ranks were then divided by the total number of genes. This generated a score between 0 (no observed mutation or variation in the gene for the given individual) and 1 (the gene has the most observed mutation or variation for the given individual) for each gene, per individual.

### Computing uEMD per gene

After rank normalization as described above, each gene has two sets of scores: one for all cancer samples and one for all healthy samples. We compare the histograms corresponding to these sets of scores using a unidirectional version of the EMD. In general, EMD is a measure of the distance between two probability distributions based on how much probability density or “dirt” must be “moved” for the two distributions to match. EMD has been used, for example, in pattern recognition contexts such as measuring the difference between two images [22]. In order to compute how often and by how much mutation scores exceed variation scores for each gene, we created a uEMD that only measures the amount of “dirt” that must be moved downward from the first distribution (mutation data) to the second (variation data) but ignores “dirt” that would be moved the other way. In practice, we compute uEMD for a gene *g* by constructing histograms for both sets of scores for that gene in 100 evenly spaced bins between 0 and 1. Then, starting from the highest bin, we count the fraction of cancer mutation scores that fall in that bin and subtract the fraction of natural variant scores that fall in that bin. Next, we move the surplus or deficit fraction of mutations to the next bin but only add any surplus to a running total for uEMD. We repeat this process for all bins or until all mutations have been accounted for. This process can equivalently be expressed by the formula

$$ uEM{D}_{\mathit{\mathsf{g}}}=\sum_{B=100}^1\max \left\{\sum_{b=100}^B\left({M}_{b,\mathit{\mathsf{g}}}-{N}_{b,\mathit{\mathsf{g}}}\right),0\right\} $$

where *M*_*b,g*_ is the fraction of mutations in bin *b* for gene *g* and *N*_*b,g*_ is the same for variants. For a fixed number of bins, computing uEMD scores for all genes is done in linear time in the number of genes.

### Test for correlation with known covariates

We tested for correlation between our per-gene uEMD scores and gene length, DNA replication time, global expression level, and chromatin state, as these covariates have been previously shown to correlate with non-silent mutation rate [8]. We computed length as the total number of bases in the longest isoform of a gene. The other three covariates were downloaded from the Cancer Genome Analysis (CGA) group [8] and were computed as described there. In each case, for each cancer type, we computed the Spearman correlation between the uEMD scores and the given measure for mutated genes.

### Evaluation

To evaluate our gene rankings, we downloaded three curated lists of known cancer genes: the list of known cancer genes in the Cancer Gene Census (CGC) from COSMIC [26], the list of “driver genes affected by subtle [point] mutations” from Vogelstein et al. [3], and the pan-cancer list of significantly mutated genes from Kandoth et al. [27]. We filtered the CGC list to only those related to somatic point mutations. We split the CGC and Vogelstein list into oncogenes and tumor suppressor genes (TSGs) as classified by each, respectively. This resulted in 202 genes in the CGC list, 47 of which are oncogenes and 52 of which are TSGs; 125 in the Vogelstein list, 54 of which are oncogenes and 71 of which are TSGs; and 137 in the Kandoth list. With respect to any list of known cancer genes, we used two methods to assess overall performance. First, since any list of known cancer genes is incomplete, we examined what fraction of top-ranking genes by our method was in the given list of genes across varying ranking cutoffs. This gave us a general idea of how enriched cancer genes were in that list. Second, to evaluate the enrichment for cancer genes across the full spectrum of predictions, we measured the area under the precision–recall curve (AUPRC) using the perfMeas package for R [28]. Note that in either case, high-scoring genes found by any method that are not in the list of known cancer genes may, in fact, correspond to newly discovered genes with functional roles in cancers. For each test, we used the list of known cancer genes as positive examples and removed known cancer genes that are implicated for other reasons from the set of negatives. Specifically, we removed all the genes we filtered out from the CGC list from the list of negatives as well as any genes that are labeled as cancer genes in any of the lists we consider. Furthermore, we removed oncogenes from the list of negatives when testing TSGs and vice versa. We applied both measures to the list of per-gene uEMD scores for each of the 24 cancer types. In evaluations against MutSigCV [8], the method developed by Youn and Simon [11], OncodriveCLUST [29], OncodriveFML [30], and MADGiC [10], we always ran these programs using default parameters on the same MAF file we used for our method. We ran FunSeq2 [19] by submitting identical MAF files to their web server using default parameters.

### Computing supporting *q*-values

To evaluate whether the uEMD of a gene is significant, we test whether it has a significantly higher uEMD than would be expected if each patient’s mutations were randomly distributed across genes. In particular, we generate randomized data as follows. First, for each individual, we randomly permute their mutation ranks across genes. Next, we use this randomized data to compute a full set of “decoy” uEMDs; that is, for each gene, we compute the uEMD between the distribution of randomized mutation ranks for that gene and its distribution of normalized variation counts across the healthy population. For each score threshold, we then compute a false discovery rate (FDR) by computing the ratio between (1) the number of decoy uEMDs at least as large as the threshold and (2) the number of genes with uEMD at least as large as the threshold when using the actual somatic mutation data. For each gene, we use its uEMD score to obtain an FDR, and a *q*-value is obtained by taking the minimum FDR for a score at least as small. This is a conservative method for controlling the FDR [23]. In practice, we repeat the randomization process five times and estimate the FDR for each gene by taking an average over these randomizations.

## Results

### Identifying cancer driver genes by differential mutation analysis

**Fig. 2:**
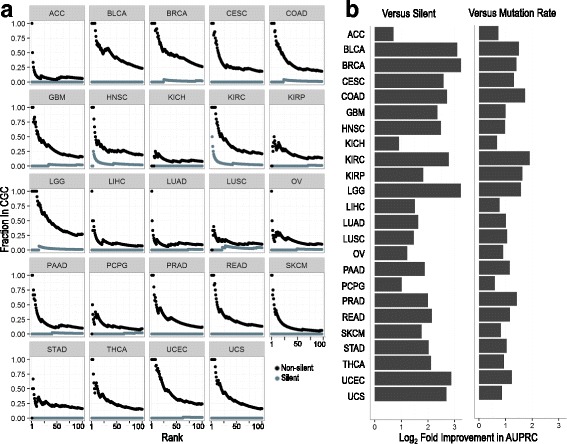
Known cancer genes are differentially mutated across 24 cancer types. **a** The fraction of genes that are in a set of known cancer driver genes [26] when we rank genes by uEMD scores as computed by DiffMut, our method for differential mutation analysis, and consider an increasing number of top-ranked genes. When computing uEMD scores using non-silent mutations, we find that a large fraction of the highest scoring genes are cancer driver genes (*black line*). When uEMD scores are computed based on silent mutations instead, we do not see an enrichment for cancer driver genes (*gray*). **b** For each cancer type, we ranked all genes by uEMD scores using either non-silent mutations or silent mutations. We then computed the log_2_ fold change in AUPRC using non-silent mutations as compared to silent mutations. As expected, AUPRCs are significantly higher when using non-silent mutations (*left*). When computing the log_2_ fold change in AUPRC when ranking genes by uEMD scores when using non-silent mutations compared to ranking them using their non-silent mutation rate, we also see a notable improvement across all cancer types (*right*).

We applied our method to all 24 cancer types sequenced in TCGA using all non-silent mutations (Additional file 1: Section A). Unlike many other methods, we do not remove hypermutated samples and do no additional pruning of genes. We evaluated our method by examining whether the CGC list of known cancer driver genes, as curated by COSMIC [26], is enriched among genes with high uEMD scores. First, since no list of known cancer genes is complete, we examined what fraction of top ranking genes by our method was in the list of known cancer genes. Across all 24 cancer types, we find that a high fraction of the top-scoring genes are, in fact, known cancer genes (Fig. 2a). Indeed, genes that are significantly differentially mutated (*q*-value < 0.1) are enriched for cancer genes (Additional file 1: Section B). As a control, we repeated this analysis using silent somatic mutations. Since silent mutations do not change protein products, we do not expect that differential mutation analysis will be predictive of cancer genes in this scenario [3]. As anticipated, we do not see an enrichment for cancer genes among genes that are the highest scoring using only silent mutation data (Fig. 2a), with only one cancer gene found with *q*-value < 0.1 across all 24 cancer types (Additional file 1: Section B).

To evaluate the enrichment for cancer genes across the full spectrum of predictions of our method, we also measured the AUPRC. To quantify the improvement in enrichment, we computed the log_2_ fold change in AUPRC between uEMD scores produced by non-silent mutations vs silent mutations (Fig. 2b, left). Next, we tested the rankings generated by our method against ranking genes by how frequently they are mutated per base of exon, a baseline method for finding cancer-related genes [12]. We found that in terms of AUPRC our method consistently outperformed mutation rate across all cancer types (Fig. 2b, right).

### Differential mutation analysis outperforms prior frequency-based methods in identifying cancer genes

**Fig. 3:**
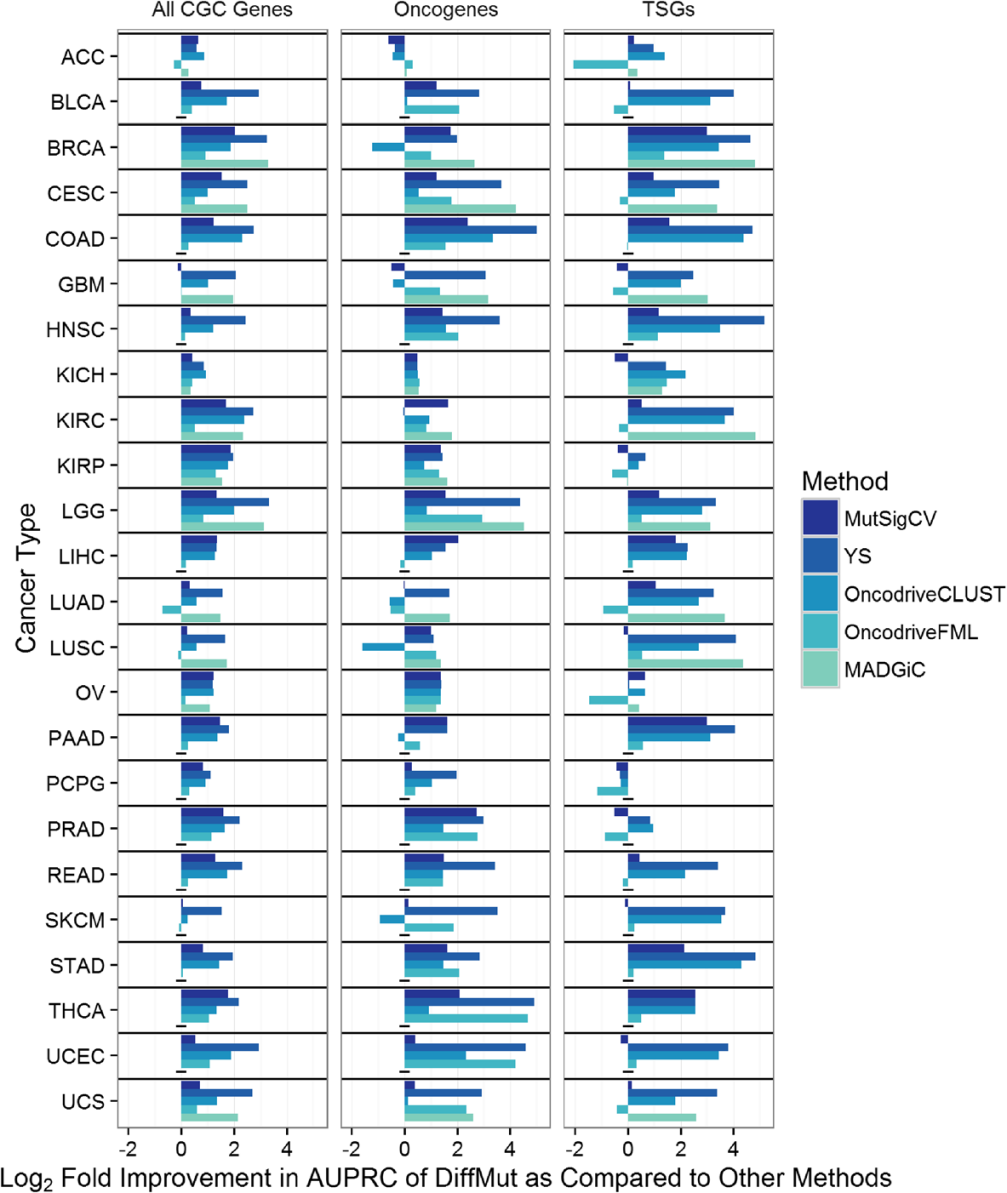
Performance of DiffMut vs other methods. The log_2_ fold change in AUPRC when ranking genes using our method, DiffMut, vs MutSigCV [8], the method developed by Youn and Simon (YS) [11], OncodriveCLUST [29], OncodriveFML [30], and MADGiC [10], when evaluating performance in identifying cancer driver genes from the Cancer Gene Census (CGC) [26] (*left*), the subset of these genes that are oncogenes (*middle*), and the subset that are TSGs (*right*). For identifying all cancer genes, differential mutation is computed based on all non-silent mutations, whereas for oncogenes and TSGs, it is computed based on only missense mutations and only nonsense mutations, respectively. Entries with a *dash* indicate cases where MADGiC could not be run

We evaluated DiffMut’s uEMD scores against gene rankings generated by MutSigCV [8], which is the de-facto standard method for detecting cancer driver genes based on somatic mutations, as well as the method developed by Youn and Simon [11], OncodriveCLUST [29], OncodriveFML [30], and MADGiC [10]. We chose these methods for evaluation because, like differential mutation analysis, they only require the user to specify a MAF file as input, in contrast to methods such as MuSiC [9], which require raw sequencing reads. Despite the relative simplicity of our method, it outperformed MutSigCV for 23 of the 24 cancer types in ranking cancer genes, as judged by AUPRC as described above (Fig. 3, left). Of particular note, DiffMut showed a fourfold improvement in AUPRC over MutSigCV in predicting cancer genes based on somatic mutations in breast cancer (BRCA). Further, DiffMut outperformed Youn and Simon’s method and OncodriveCLUST in all 24 cancer types, MADGiC on all 12 types we could run that program on, and OncdodriveFML on 19. Overall, we dominate most competing methods over the full length of the precision recall curve, both on the 24 individual cancers and in pan-cancer analysis (Additional file 1: Section C).

We also performed several other evaluations of our method. First, we tested the log_2_ fold change in AUPRC of DiffMut vs the other methods up to only 10% recall; we obtained similar results, suggesting good performance in the top range of predictions (Additional file 1: Section D). Second, we considered the cancer-specific driver genes identified in the CGC; while these sets of genes are too small for meaningful AUPRC computations, we found that for each cancer type, the cancer-specific genes were generally ranked higher than other known cancer genes (Additional file 1: Section E). This implies that DiffMut preferentially selects cancer-specific genes rather than repeatedly identifying the same set of genes across cancer types. Third, we evaluated our method on the curated lists of cancer genes described by Vogelstein et al. [3] and Kandoth et al. [27] and obtained similar results (Additional file 1: Section F). Fourth, we performed runtime analysis of our method and found that it is typically significantly faster than previous approaches; for example, when run on the BRCA dataset, DiffMut is 30 times faster than MutSigCV, even when run on a less powerful machine (Additional file 1: Section G). Finally, we confirmed that uEMD scores do not correlate with known covariates (Additional file 1: Section H). We conclude our general evaluation of how well DiffMut identifies known cancer genes by noting that the performance of all these methods, including our own, can likely be improved by additional curation and processing [31]; however, our goal was to perform an automated, large-scale comparative analysis on identical mutation files without any further optimizations or gene or patient pruning.

### Differential mutation analysis can separately identify oncogenes and tumor suppressor genes

The list of known cancer genes from the Cancer Gene Census is divided into oncogenes and TSGs, due to the well-established significant biological differences between the two. While oncogenes drive cancer growth with specific functional mutations, TSGs inhibit growth when functioning normally. It is therefore thought that TSGs can be easily disrupted by nonsense mutations [3]. Because of this fundamental biological difference between TSGs and oncogenes, we decided to analyze missense and nonsense mutations separately. As expected, when using only missense mutations, we are better able to predict oncogenes; and when using only nonsense mutations, we are much better able to predict TSGs. The vast majority of the time, our method is better able to detect oncogenes and TSGs than the five methods to which we compare (Fig. 3 middle and right). We see similar results using the set of oncogenes and TSGs described by Vogelstein et al. (Additional file 1: Section F). Thus, our approach allows us to enrich for specific subtypes of cancer driver genes while other methods have not been shown to readily make this distinction.

### Differential mutation analysis reveals that many long genes with high mutation rates in cancers are also highly variable across natural populations

Olfactory receptors and some extraordinarily long genes (including the muscle protein *TTN*, the membrane associated mucins *MUC4* and *MUC16*, and the nuclear envelope spectrin-repeat protein *SYNE1*) have high mutation rates, but it has been proposed that mutations within them are unlikely to play causal roles in cancers [8]. In support of this, of the 372 olfactory receptor genes found in the HORDE database [32], none are found to be significantly differentially mutated (*q*-value < 0.1) in 23 of the 24 cancer types we analyzed, and only one is found to be differentially mutated in the last cancer type. In contrast, the five other tested methods often do not show the same under enrichment for olfactory receptor genes among their lists of predicted driver genes (Additional file 1: Section I). Similarly, of the ten longest genes with above average mutation rates, none are implicated by differential mutation across any of the 24 cancer types (Additional file 1: Section I). That is, while these genes have a high mutation rate for their length, they also vary naturally at a higher rate. Although the functions of some of these genes are not fully known, and some may, in fact, be cancer related, their relationship to the disease is likely complex and so they are not expected to be implicated by somatic mutation alone [8]. Thus, differential mutational analysis provides a powerful yet simple approach to eliminate genes that have high somatic mutation rates but are found to be highly variable across human populations.

### Differential mutation analysis proposes new cancer driver genes

**Fig. 4:**
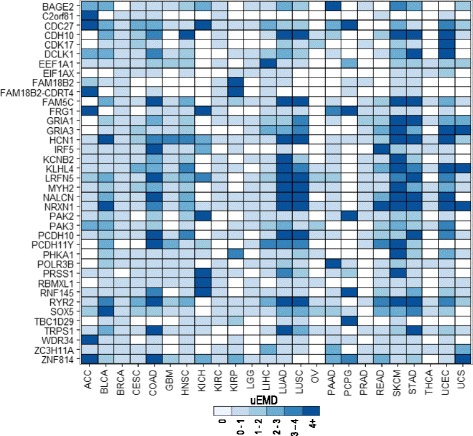
Genes that are proposed cancer drivers by differential mutation. Shown are all genes that are among the five most significantly differentially mutated genes for any given cancer that are not already known cancer driver genes. Genes that show no differential mutation in a given cancer have a uEMD score of 0 and are in *white*. All genes with a uEMD score greater than 0 showed some level of differential mutation and are shown in shades of *blue* with increasing intensity

Although many of the genes found to be differentially mutated are known cancer genes, high-scoring genes not in the list of known cancer genes may, in fact, correspond to newly discovered genes with functional roles in cancers. For example, two genes that we found to be significantly differentially mutated, *TRPS1* and *ZNF814*, both contain numerous mutations in and near their DNA-binding zinc finger domains. Across all the samples in TCGA, we observed 103 missense mutations of a single nucleotide in *ZNF814*, indicating that it may be an oncogene by the definition presented in Vogelstein et al. [3]. *TRPS1*, on the other hand, contains 18 nonsense and 228 missense mutations across its exons, suggesting that it may be a TSG. It has previously been reported that *TRPS1* plays a role in cancer development [33], and that higher levels of *TRPS1* improved survival [34]. Similarly, *CDH10* contains 20 nonsense and 319 missense mutations and, in agreement with our results, has previously been identified as a potential TSG in colorectal cancer and lung squamous cell carcinoma [35, 36]. Other differentially mutated genes such as *EIF1AX* have been reported by previous studies [37, 38] but are absent from the gold standards we used. A full list of genes that were not already included in our lists of positives but show significant differential mutation across the 24 cancer types can be found in Fig. 4.

## Discussion

We have shown that natural germline variation data serve as a powerful source of information for discovering cancer driver genes. This one type of data allowed us to develop a fast (Additional file 1: Section G) and simple non-parametric method for detecting cancer driver genes with higher precision than currently used methods without the use of any extraneous covariate data. In the future, alternate approaches to uncover genes differentially mutated between cancer and healthy cohorts can be developed based upon the increasing availability of data and may yield even better performance. Encouragingly, we observe that the power of our current differential mutation analysis method increases as more tumor samples are sequenced (Additional file 1: Section G), thereby suggesting that further cancer genome sequencing will increase the predictive power of our framework.

As larger numbers of healthy human genomes are sequenced and germline variation data become more abundant, our approach can likely be improved via explicit modeling of population structure. Indeed, many variant sites may be stable within subpopulations. For example, sub-Saharan African populations exhibit a great deal of natural variation relative to European populations [39]. Ashkenazi Jewish populations, on the other hand, show less genetic variation [40] and, significantly, show genetic predisposition to some types of cancer [41]. In order to account for this, in the future, variants could be counted only when they differ within the appropriate subpopulation.

Another benefit of further sequencing would be an increase in the density of observed mutations and variants. Currently, there are only enough data to glean differential mutation on a whole-gene level. However, with denser annotation it may be possible to score smaller regions of genes such as known functional domains. For example, HLA genes, which are highly variable, all have very low differential mutation scores. However, much of this is due to natural variation within specific genic regions. In the future, it may be possible to evaluate regions such as these separately to determine whether mutations in other less variable parts of genes are important in cancers.

While this work introduces the idea of detecting cancer-relevant genes by identifying those that are differentially mutated between cancer cohorts and healthy populations, natural variation has previously been used to measure the impact of specific mutations. Cancer mutations that fall directly onto variant sites are often discarded [12] and some somatic mutations that fall into regions with a high ratio of rare variants to common ones can have a large functional impact [18]. Previous approaches have aimed to find such mutations across patients with the goal of identifying mutations that drive each patient’s cancer [19]. Although these previous approaches are not designed to identify cancer driver genes and do not perform well at this task (Additional file 1: Section F), identifying driver mutations is a challenging parallel task and a potential direction for further work with differential mutation analysis.

Thus far, we have only shown the power of differential mutation in identifying individual genes that may play a role in cancer. However, it is well understood that cancer is a disease of pathways [3, 4]. Thus, an especially promising avenue for future work is in performing differential mutation analysis at the pathway level. In particular, gene-set and pathway analyses can be performed by examining how germline variation accumulates across entire sets of genes and assessing whether there is evidence of differential mutation at that level as well. Differential mutation analysis could also potentially be integrated into network-based approaches that do not require known pathway annotations but instead uncover novel cancer pathways [42, 43].

Finally, similar to other methods for detecting cancer driver genes, differential mutation analysis is likely to benefit from domain-specific knowledge. For example, in melanomas there are a large number of C to T mutations that are the result of ultraviolet radiation [6, 8]. Because these mutations occur in a much higher abundance than other mutations, they dominate the mutational signal. We therefore hypothesize that it may be beneficial to look at specific types of mutations for some cancers. Further improvements on other cancer types are also likely to be possible by explicitly considering mutational context. Similarly, in cancer types where non-point mutations (such as copy number variation, insertions, or deletions) play a larger role than somatic mutation, incorporating additional knowledge on these mutation types from both cancer and natural variation data will broaden our ability to predict cancer-related genes.

## Conclusions

Despite somatic mutations and germline variants being subject to a different set of evolutionary pressures [7], we propose that genes observed to have numerous variants across the population are able to accumulate more somatic mutations without experiencing a drastic functional change. While we presented a method that directly leverages this idea and have shown that it is highly effective in identifying cancer-related genes, it is likely that even more powerful predictors of cancer driver genes could be obtained by integrating natural variation data with other information. In conclusion, we propose that akin to the prominent role of differential expression analysis in analyzing cancer expression datasets, differential mutation analysis is a natural and powerful technique for examining genomic alteration data in cancer studies.

## Additional file

Additional file 1: A file containing all additional figures and tables. **Section A** shows the total mutation counts for all the cancer types we analyzed. **Section B** shows the enrichment for cancer genes among differentially mutated genes when using non-silent mutations but not when using silent mutations. **Section C** shows the full precision–recall curves and the areas under them. **Section D** shows the log-fold change in AUPRC when only computing the area up to 10% recall. **Section E** shows how known cancer-specific genes are ranked relative to all known cancer genes. **Section F** shows the performance of our method against other methods when evaluated using several different lists of known cancer genes. **Section G** shows the fast runtime of our method and how the power of our method increases with more tumor samples. **Section H** shows that our method’s ranking of genes does not correlate with known covariates. **Section I** shows DiffMut’s lack of enrichment for olfactory receptors and extraordinarily long genes among its top-ranked genes. (PDF 1097 kb)

## Abbreviations

AUPRC: Area under the precision–recall curve
CGC: Cancer Gene Census
TCGA: The Cancer Genome Atlas
TSG: Tumor suppressor gene
uEMD: Unidirectional Earth Mover’s Distance
