# Correction to: Differential analysis between somatic mutation and germline variation profiles reveals cancer-related genes

**DOI:** 10.1186/s13073-018-0544-3

**Published:** 2018-05-10

**Authors:** Pawel F. Przytycki, Mona Singh

**Affiliations:** 1 Department of Computer Science, Princeton University, Princeton, NJ 08544 USA; 2 Lewis-Sigler Institute for Integrative Genomics, Princeton University, Princeton, NJ 08544 USA

## Correction

Due to an error introduced during copyediting of this article [1], reference [8] incorrectly reads:

“Noble M, DiCara D, Lin P, Lichtenstein L, Heiman DI, Fennell T, et al. Mutational heterogeneity in cancer and the search for new cancer-associated genes. Nature. 2013;499:214–8. 10.1038/nature12213.”

The correct reference [8] should instead read:

“Lawrence MS, Stojanov P, Polak P, Kryukov GV, Cibulskis K, Sivachenko A, et al. Mutational heterogeneity in cancer and the search for new cancer-associated genes. Nature. 2013;499:214–8. 10.1038/nature12213.”
