# Supplementary material for: Differential analysis between somatic mutation and germline variation profiles reveals cancer-related genes
Source: Genome Med. 2017 Aug 25;9:79. doi: 10.1186/s13073-017-0465-6 (PMC5574113; doi:10.1186/s13073-017-0465-6)
Supplement: Additional file 1: — A file containing all additional figures and tables. Section A shows the total mutation counts for all the cancer types we analyzed. Section B shows the enrichment for cancer genes among differentially mutated genes when using non-silent mutations but not when using silent mutations. Section C shows the full precision–recall curves and the areas under them. Section D shows the log-fold change in AUPRC when only computing the area up to 10% recall. Section E shows how known cancer-specific genes are ranked relative to all known cancer genes. Section F shows the performance of our method against other methods when evaluated using several different lists of known cancer genes. Section G shows the fast runtime of our method and how the power of our method increases with more tumor samples. Section H shows that our method’s ranking of genes does not correlate with known covariates. Section I shows DiffMut’s lack of enrichment for olfactory receptors and extraordinarily long genes among its top-ranked genes. (PDF 1097 kb) [file 13073_2017_465_MOESM1_ESM.pdf]

Additional figures and tables

Section A shows the total mutation counts for all the cancer types we analyzed. **Section B** shows the enrichment for cancer genes amongst differentially mutated genes when using non-silent mutations but not when using silent mutations. **Section C** shows the full precision-recall curves and the areas under them. **Section D** shows the log-fold change in AUPRC when only computing the area up to 10% recall. **Section E** shows how known cancer-specific genes are ranked relative to all known cancer genes. **Section F** shows the performance of our method against other methods when evaluated using several different lists of known cancer genes. **Section G** shows the fast runtime of our method and how the power of our method increases with more tumor samples. **Section H** shows that our method's ranking of genes does not correlate with known covariates. **Section I** shows DiffMut's lack of enrichment for olfactory receptors and extraordinarily long genes amongst its top ranked genes.

Section A: Summary of TCGA mutation counts across cancer types

This table is a summary of the numbers of samples and the total numbers of each mutation type we observed when mapping data from TCGA [1].

Cancer Type		Number of Samples	Number of Mutations		
			Missense	Nonsense	Silent
Adrenocortical carcinoma	ACC	90	10,734	582	6,265
Bladder urothelial carcinoma	BLCA	237	48,490	4,658	19,272
Breast invasive carcinoma	BRCA	987	50,067	4,088	18,510
Cervical squamous cell carcinoma and endocervical adenocarcinoma	CESC	193	25,060	2,515	9,347
Colon adenocarcinoma	COAD	216	62,620	4,359	32,045
Glioblastoma multiforme	GBM	291	13,746	833	5,335
Head and neck squamous cell carcinoma	HNSC	509	64,988	5,026	26,169
Kidney chromophobe	KICH	66	4,212	177	1,922
Kidney renal clear cell carcinoma	KIRC	417	16,369	1,134	6,239
Kidney renal papillary cell carcinoma	KIRP	168	12,752	1,151	5,056
Brain lower grade glioma	LGG	220	16,403	1,057	6,503
Liver hepatocellular carcinoma	LIHC	202	18,222	1,088	7,042
Lung adenocarcinoma	LUAD	543	125,750	9,967	44,406
Lung squamous cell carcinoma	LUSC	176	42,280	3,685	15,654
Ovarian serous cystadenocarcinoma	OV	141	4,262	279	1,421
Pancreatic adenocarcinoma	PAAD	143	23,382	1,527	9,777
Pheochromocytoma and paraganglioma	PCPG	184	3,164	79	786
Prostate adenocarcinoma	PRAD	261	8,984	512	3,691
Rectum adenocarcinoma	READ	80	15,713	1,690	5,866
Skin cutaneous melanoma	SKCM	367	169,416	10,477	96,740
Stomach adenocarcinoma	STAD	279	86,726	4,361	36,256
Thyroid carcinoma	THCA	404	4,458	246	1,692
Uterine corpus endometrial carcinoma	UCEC	247	116,388	12,379	41,478
Uterine carcinosarcoma	UCS	57	5,318	523	1,738

Section B: Enrichment for cancer genes

Genes in the Cancer Gene Census (CGC) [2] are enriched among differentially mutated genes when computing the uEMD based on non-silent mutations. As a control, we also computed differential mutation based on silent mutations (which are not expected to be predictive of cancer genes). We computed *p*-values based on the hypergeometric distribution to test whether there are more CGC genes than expected by chance amongst the set of significantly differentially mutated genes (*q*-value < .1).

	**Number of CGC Genes (*p*-value)**	
Type	Non-silent Mutations	Silent Mutations
ACC	1 (1.269E-01)	0 (1.000E+00)
BLCA	17 (4.695E-26)	0 (1.000E+00)
BRCA	11 (1.509E-19)	0 (1.000E+00)
CESC	5 (6.825E-10)	0 (1.000E+00)
COAD	11 (2.110E-15)	0 (1.000E+00)
GBM	7 (7.018E-11)	0 (1.000E+00)
HNSC	17 (2.040E-19)	0 (1.000E+00)
KICH	1 (7.045E-02)	0 (1.000E+00)
KIRC	6 (1.162E-12)	1 (1.038E-02)
KIRP	2 (1.050E-03)	0 (1.000E+00)
LGG	9 (1.160E-17)	0 (1.000E+00)
LIHC	2 (4.567E-03)	0 (1.000E+00)
LUAD	16 (3.679E-10)	0 (1.000E+00)
LUSC	9 (2.201E-07)	0 (1.000E+00)
OV	1 (1.038E-02)	0 (1.000E+00)
PAAD	5 (1.839E-05)	0 (1.000E+00)
PCPG	4 (5.873E-05)	0 (1.000E+00)
PRAD	2 (1.072E-04)	0 (1.000E+00)
READ	6 (3.254E-09)	0 (1.000E+00)
SKCM	16 (2.172E-08)	0 (1.000E+00)
STAD	16 (3.806E-13)	0 (1.000E+00)
THCA	3 (4.375E-06)	0 (1.000E+00)
UCEC	23 (2.177E-24)	0 (1.000E+00)
UCS	8 (1.444E-13)	0 (1.000E+00)

Section C: Full precision-recall curves and AUPRCs

To evaluate the performance of our method relative to the others we used the area under the precision-recall curve. For these curves we evaluated the performance of DiffMut against MutSigCV [3], the method developed by Youn and Simon (YS) [4], OncodriveCLUST [5], OncodriveFML [6], and MADGiC [7] on the list of genes from the CGC [2]. Shown here are (1) the full precision-recall curves for all 24 cancer types individually and for combined data and (2) the areas computed under those curves (AUPRC) with the highest AUPRC for each cancer type bolded. Combined analysis was performed in two ways: (1) aggregation of the results from cancer-specific analyses and (2) pan-cancer, where each method is run once on all samples from all cancer types. To aggregate cancer-specific data, for each gene, we assigned to it the minimum *q*-value (or *p*-value in the case of MADGiC and YS) found for it across all cancer types. Genes with identical minimum significance values were ordered by counting how many different cancer types each gene was found significant in at that threshold, normalized by how many genes were significant in that cancer at that threshold. Pan-cancer analysis was performed by running each method directly on a concatenation of all samples resulting in a single MAF file with 3,247,379 mutations across 12,224 samples; methods that did not terminate are not shown in the PR-curves and are indicated by a dash in the table of AUPRCs. Note that our method generally dominates other methods across the full length of the curve, showing that it has higher precision across most recalls.

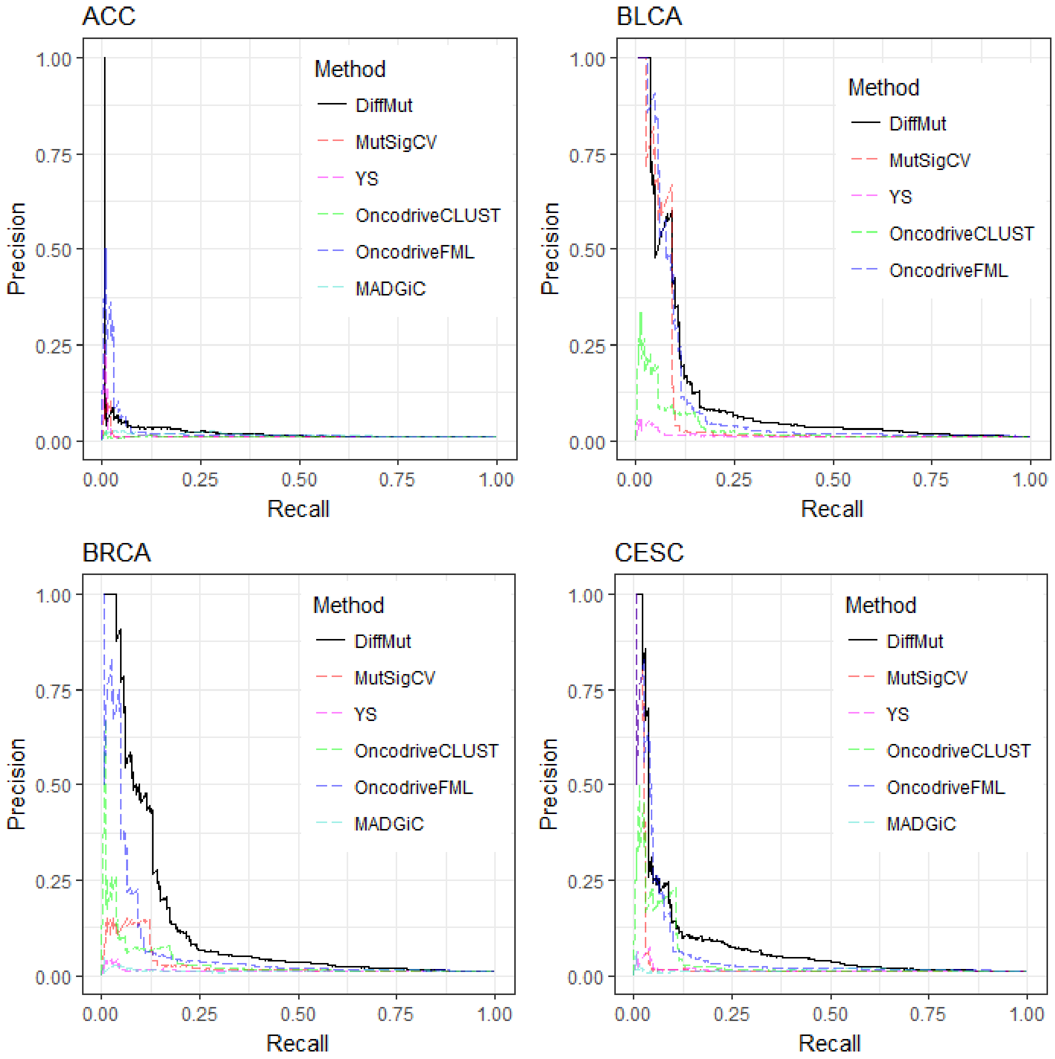

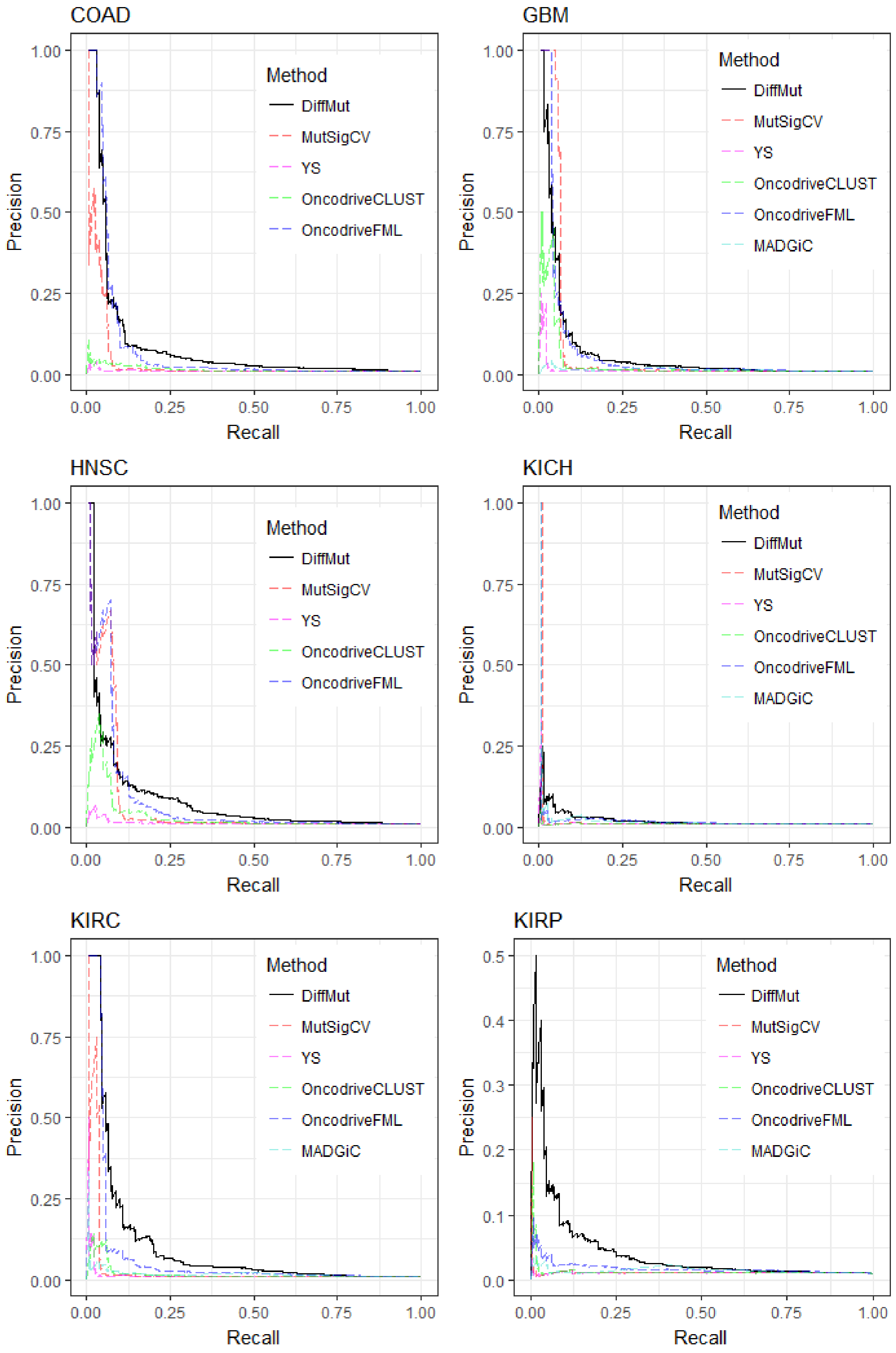
COAD
Precision
Recall
0.00
0.25
0.50
0.75
1.00
Method
DiffMut
MutSigCV
YS
OncodriveCLUST
OncodriveFML
GBM
Precision
Recall
Method
DiffMut
MutSigCV
YS
OncodriveCLUST
OncodriveFML
MADGiC
HNSC
Precision
Recall
Method
DiffMut
MutSigCV
YS
OncodriveCLUST
OncodriveFML
KICH
Precision
Recall
Method
DiffMut
MutSigCV
YS
OncodriveCLUST
OncodriveFML
MADGiC
KIRC
Precision
Recall
Method
DiffMut
MutSigCV
YS
OncodriveCLUST
OncodriveFML
MADGiC
KIRP
Precision
Recall
0.0
0.1
0.2
0.3
0.4
0.5
Method
DiffMut
MutSigCV
YS
OncodriveCLUST
OncodriveFML
MADGiC

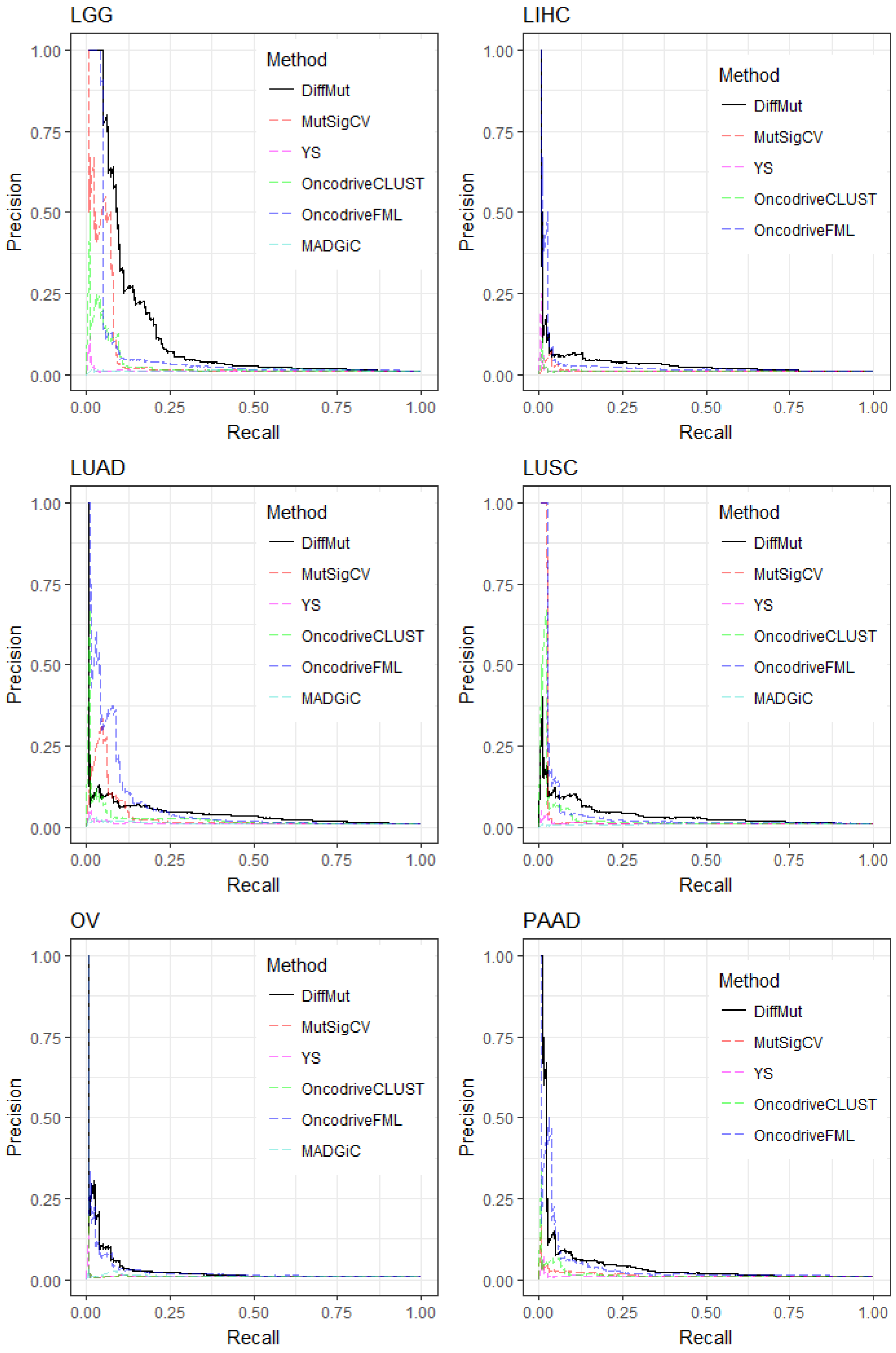
LGG
LIHC
LUAD
LUSC
OV
PAAD
Precision
Recall
Method
DiffMut
MutSigCV
YS
OncodriveCLUST
OncodriveFML
MADGiC
0.00
0.25
0.50
0.75
1.00

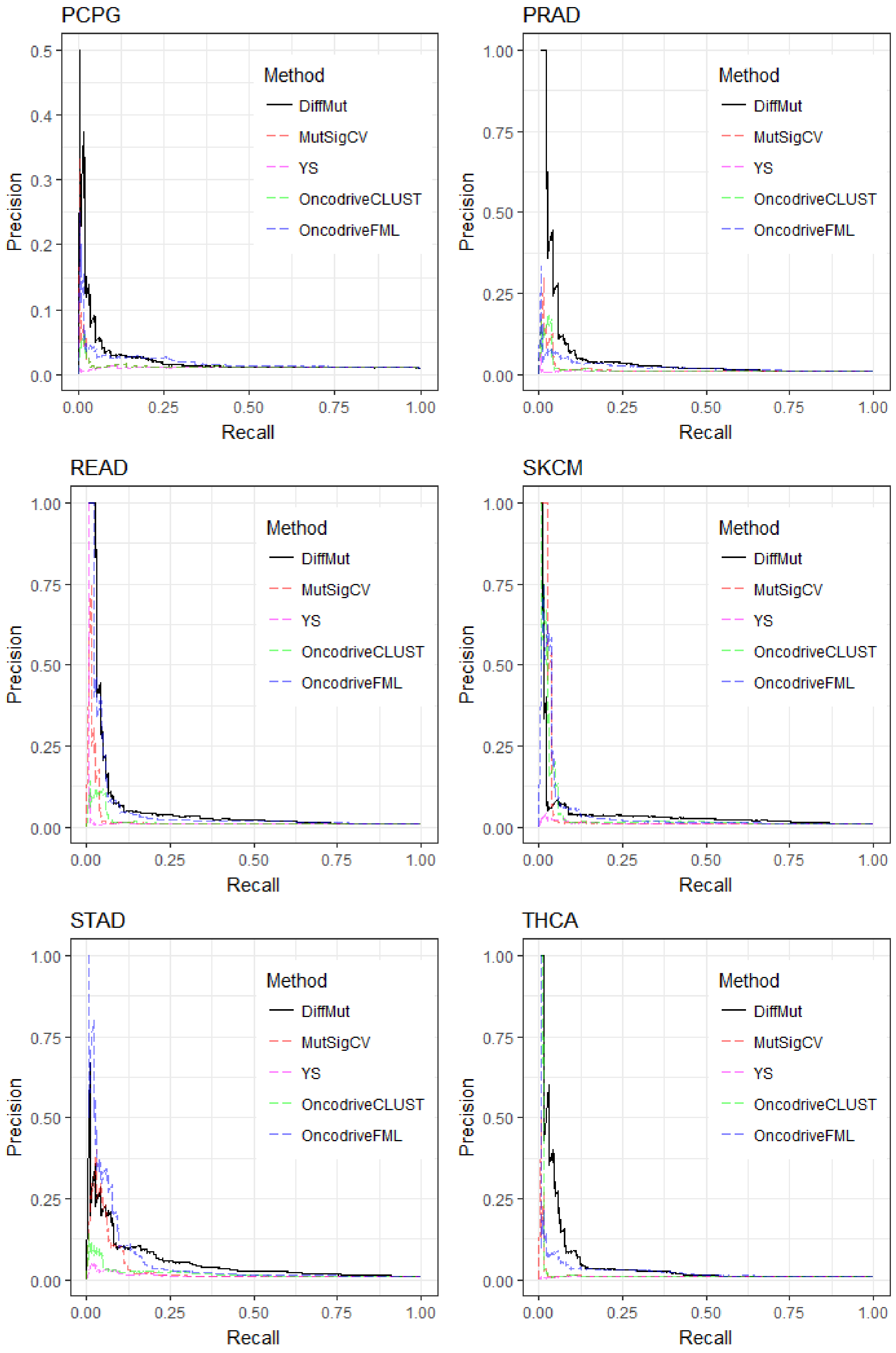
PCPG
PRAD
READ
SKCM
STAD
THCA
Precision
Recall
Method
DiffMut
MutSigCV
YS
OncodriveCLUST
OncodriveFML
0.0
0.1
0.2
0.3
0.4
0.5
0.00
0.25
0.50
0.75
1.00

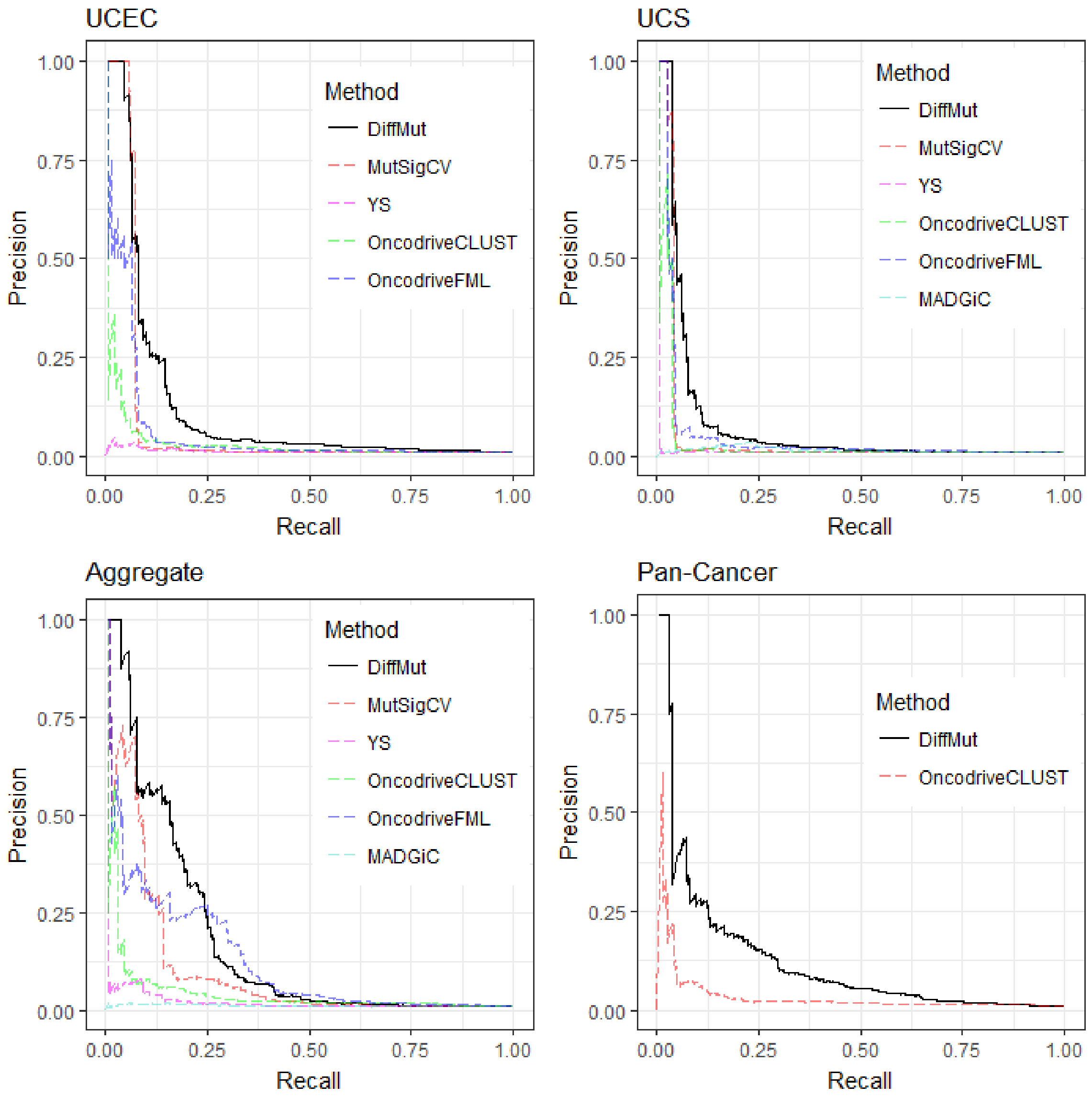

UCEC
UCS
Aggregate
Pan-Cancer
Method
DiffMut
MutSigCV
YS
OncodriveCLUST
OncodriveFML
MADGiC
Precision
Recall
0.00
0.25
0.50
0.75
1.00

Type	AUPRC					
	DiffMut	MutSigCV	YS	MADGiC	OncodriveCLUST	OncodriveFML
ACC	0.020217	0.013017	0.01357	0.01598	0.011107	**0.02512**
BLCA	**0.107072**	0.078411	0.014235	-	0.031544	0.096057
BRCA	**0.124818**	0.028921	0.01304	0.012525	0.033374	0.066298
CESC	**0.079032**	0.027838	0.01404	0.013695	0.039019	0.056142
COAD	**0.084149**	0.033806	0.012269	-	0.016348	0.076517
GBM	0.063135	**0.06999**	0.015027	0.015694	0.031289	0.062791
HNSC	**0.075641**	0.063878	0.013924	-	0.032961	0.074032
KICH	**0.021478**	0.01648	0.012071	0.016378	0.011413	0.016424
KIRC	**0.096528**	0.030047	0.014624	0.018559	0.018532	0.06816
KIRP	**0.042787**	0.011865	0.011114	0.015032	0.012791	0.01773
LGG	**0.123032**	0.049121	0.012271	0.013652	0.02928	0.069855
LIHC	**0.031034**	0.012419	0.012357	-	0.01204	0.02813
LUAD	0.038316	0.031443	0.012779	0.012998	0.025604	**0.064277**
LUSC	0.038167	0.031071	0.012195	0.011547	0.026172	**0.042839**
OV	**0.026646**	0.011239	0.011584	0.012674	0.011245	0.024334
PAAD	**0.042304**	0.015277	0.012027	-	0.016104	0.03614
PCPG	**0.023793**	0.013647	0.010988	-	0.012631	0.019535
PRAD	**0.051689**	0.017565	0.011308	-	0.016652	0.023966
READ	**0.058281**	0.023862	0.011684	-	0.017364	0.048844
SKCM	0.037887	0.03718	0.013181	-	0.032443	**0.040922**
STAD	**0.054811**	0.032509	0.014232	-	0.020261	0.052524
THCA	**0.050264**	0.014767	0.010991	-	0.020077	0.024394
UCEC	**0.112073**	0.075816	0.0139	-	0.028019	0.054362
UCS	**0.073228**	0.045301	0.011225	0.015731	0.028864	0.048823
Aggregate	**0.168499**	0.097205	0.020050	0.012653	0.041374	0.119033
Pan-cancer	**0.117466**	-	-	-	0.034791	-

Section D. Performance of DiffMut as compared to other methods up to 10% recall

We evaluated the performance of DiffMut against MutSigCV [3], the method developed by Youn and Simon (YS) [4], OncodriveCLUST [5], OncodriveFML [6], and MADGiC [7] when using the list of cancer driver genes from CGC [2]. We computed the $\log_2$ fold change in AUPRC between our method and the given one when judged on ranking all cancer driver genes (left), oncogenes (middle), and tumor suppressor genes (TSGs, right) but only up to a 10% recall. This only compares the relative performances of the methods at high specificity. Entries with a dash indicate cases where MADGiC could not be run.

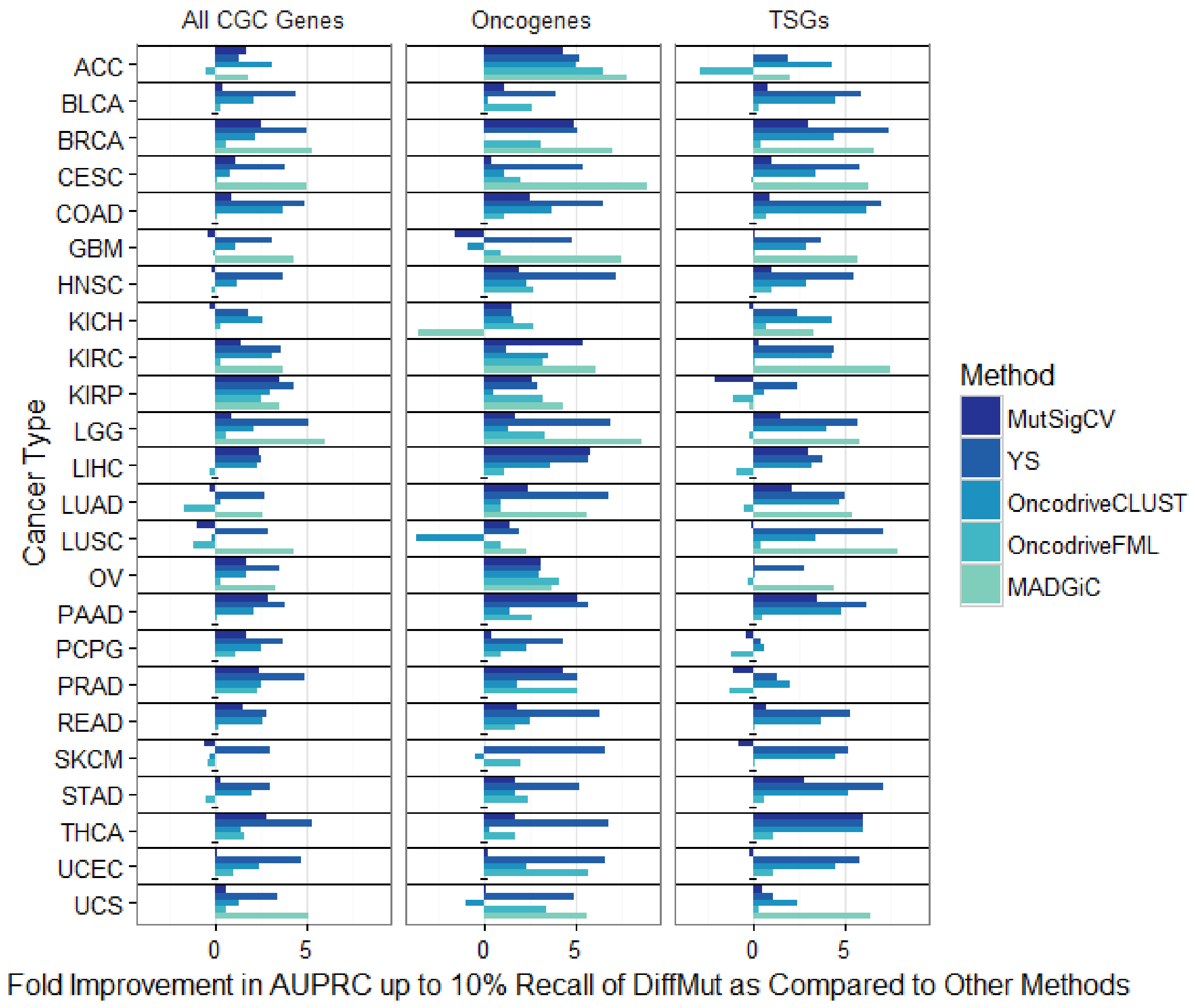

Section E: **Known cancer-specific genes tend to be ranked higher than other known cancer genes**

For each cancer type, we collected a subset of genes from the Cancer Gene Census that were labeled as relevant to that cancer type. This resulted in no genes for some cancer types and as many as 30 for others. We note that these are too small sets of positives to generate informative precision-recall curves. Shown are the rankings, by uEMD, for the given cancer type of cancer-specific genes (right) versus all other known cancer genes from the Cancer Gene Census (left). The cancer-specific genes are generally ranked higher indicating that DiffMut is preferentially selecting cancer-specific genes rather than repeatedly choosing the same set of cancer genes (cancer types where the difference is statistically significant at $p<0.05$ by a one-sided Wilcoxon test are marked with an asterisk).

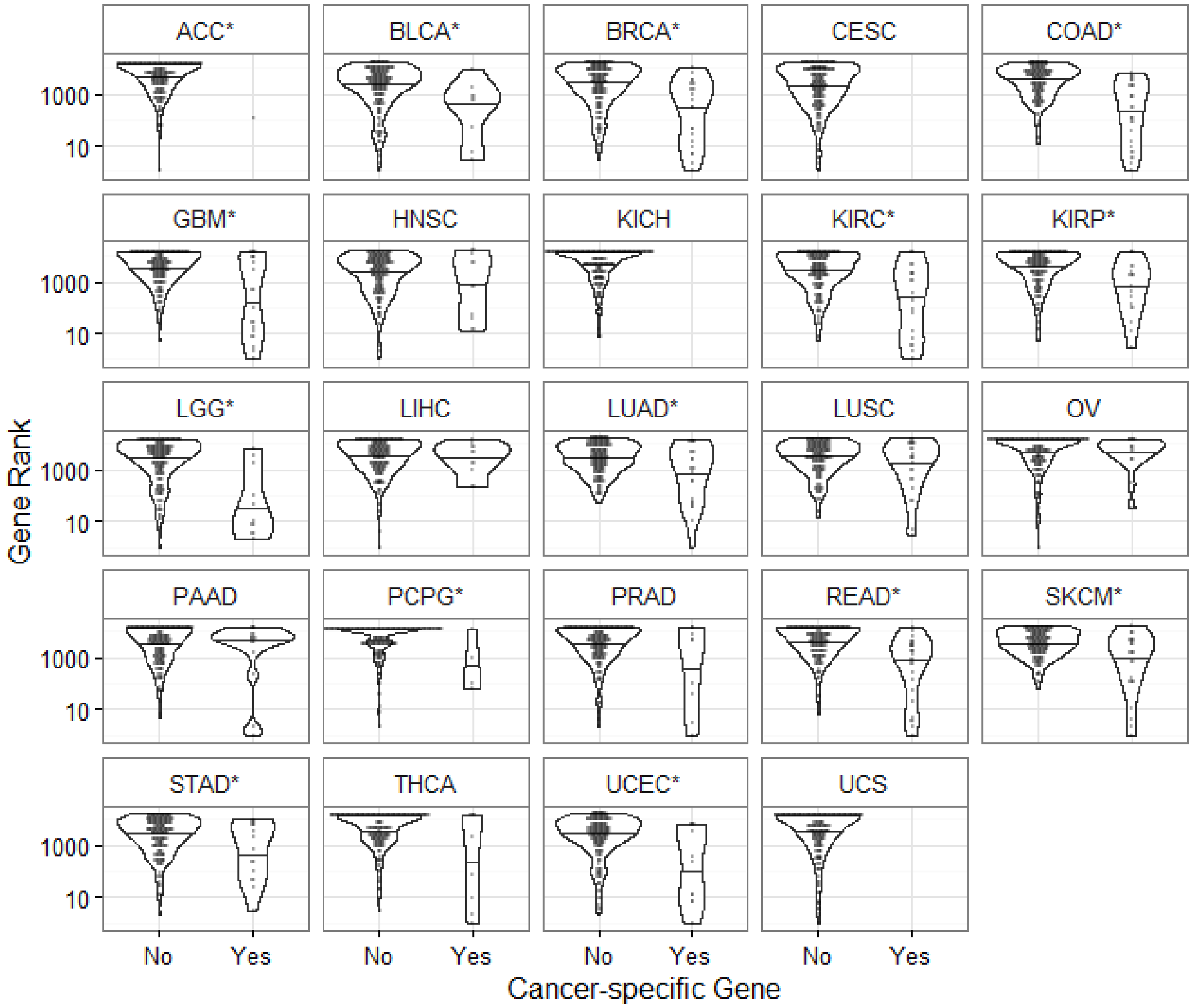

Section F: Performance of DiffMut as compared to other methods, and using different sources of known cancer genes

We evaluated the performance of DiffMut against MutSigCV [3], the method developed by Youn and Simon (YS) [4], OncodriveCLUST [5], OncodriveFML [6], and MADGiC [7] when using the list of cancer driver genes from Vogelstein et. al [8] as our gold standard. We computed the $\log_2$ fold change in AUPRC between our method and the given one when judged on ranking all cancer driver genes (left), oncogenes (middle), and tumor suppressor genes (TSGs, right). Entries with a dash indicate cases where MADGiC could not be run.

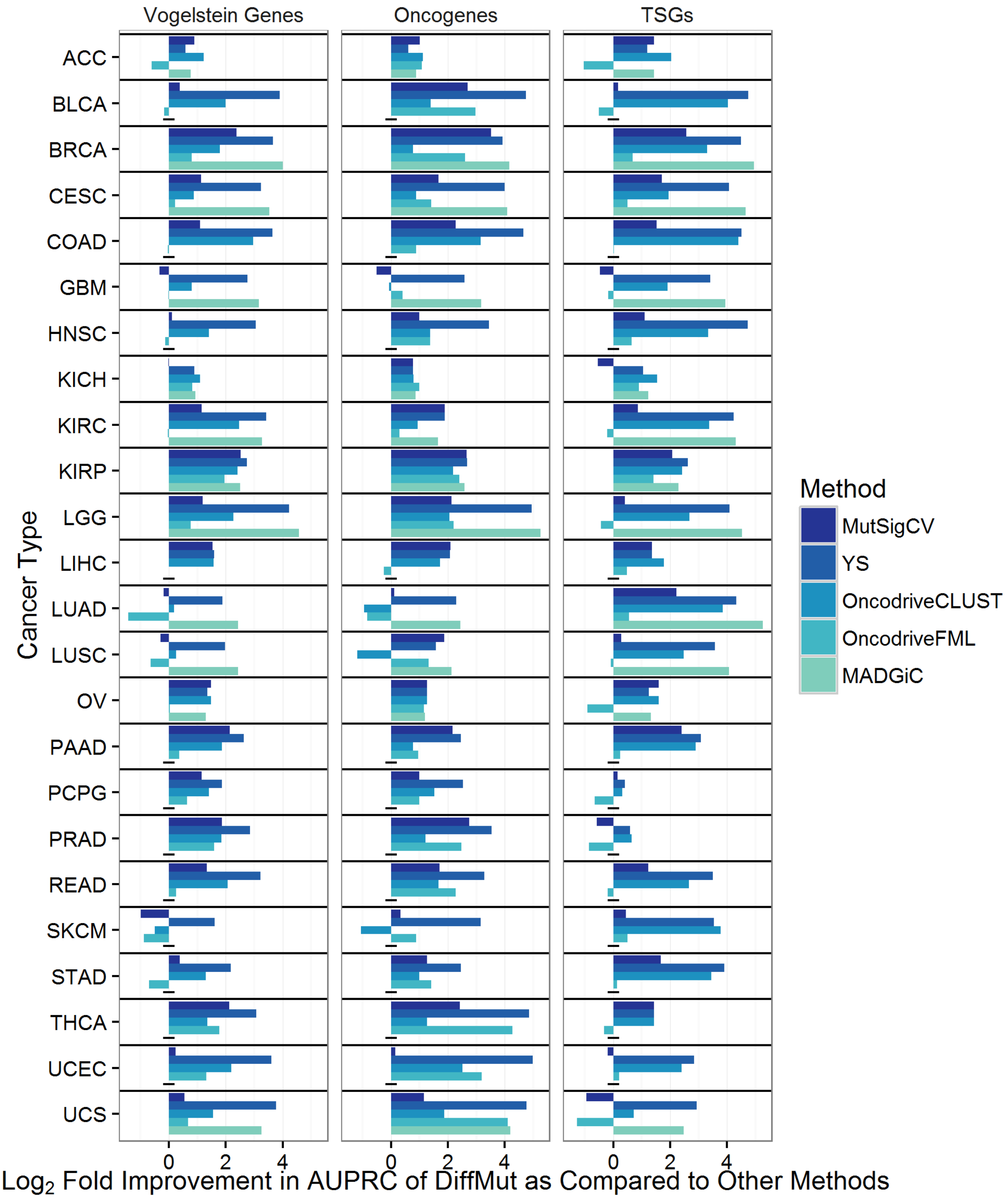

Similarly, we evaluated the performance of our method compared against all five competing methods on the list of cancer driver genes from Kandoth et. all [9]. Entries with a dash indicate cases where MADGiC could not be run.

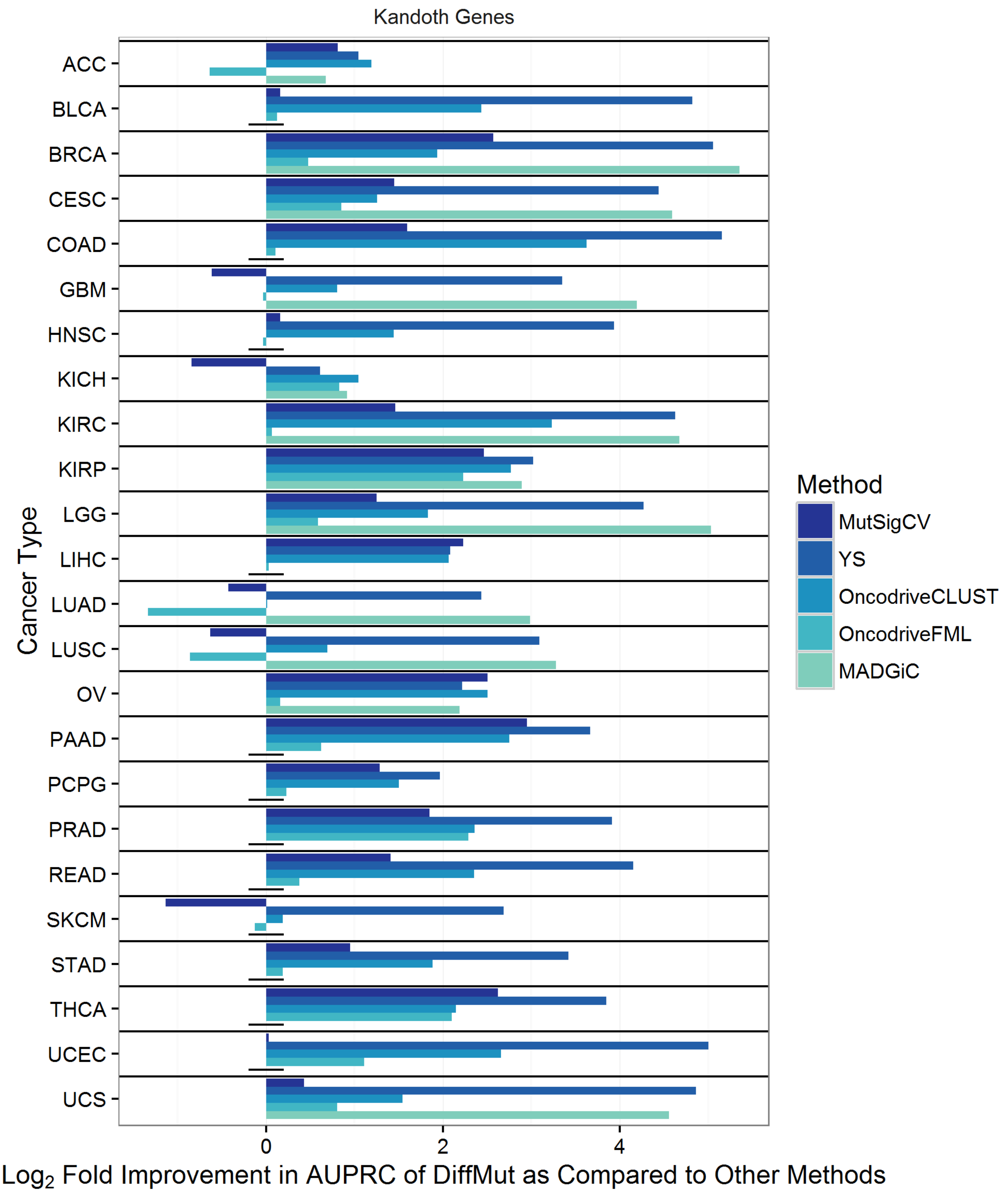

Among many other features, FunSeq2 [10] finds somatic mutations that fall into regions with a high ratio of rare variants to common ones, with the goal of identifying mutations that drive each patient's cancer. As a test of its ability to predict cancer genes, we ran FunSeq2 on the same MAF files as we used for our method and tested if the genes containing the somatic mutations deemed significant were enriched in CGC genes (hypergeometric test). We found that these genes were only moderately enriched in cancer driver genes (in the cases of STAD and UCEC, the method never terminated).

Type	Number of CGC Genes (p-value)
ACC	1 (0.27)
BLCA	2 (0.38)
BRCA	0 (1.00)
CESC	1 (0.52)
COAD	1 (0.34)
GBM	5 (1.00E-5)
HNSC	6 (1.34E-3)
KICH	1 (0.42)
KIRC	2 (0.12)
KIRP	0 (1.00)
LGG	2 (7.76E-3)
LIHC	3 (0.12)
LUAD	4 (0.10)
LUSC	3 (0.38)
OV	5 (2.00E-4)
PAAD	5 (0.04)
PCPG	1 (0.16)
PRAD	2 (0.19)
READ	4 (2.54E-3)
SKCM	3 (0.04)
STAD	- (-)
THCA	1 (0.24)
UCEC	- (-)
UCS	2 (0.09)

Section G: Runtime and Power Analysis

Our method performs differential mutation analysis for each of the 24 cancer types within minutes, even for large studies with nearly 1,000 samples. Running time increases linearly with the number of tumor samples. Timing is based on runs with default settings in which five background distributions are generated and does not include the time it takes to read from disk. We tested the code on a desktop computer using a single core running at 3.4Ghz with 8GB of RAM.

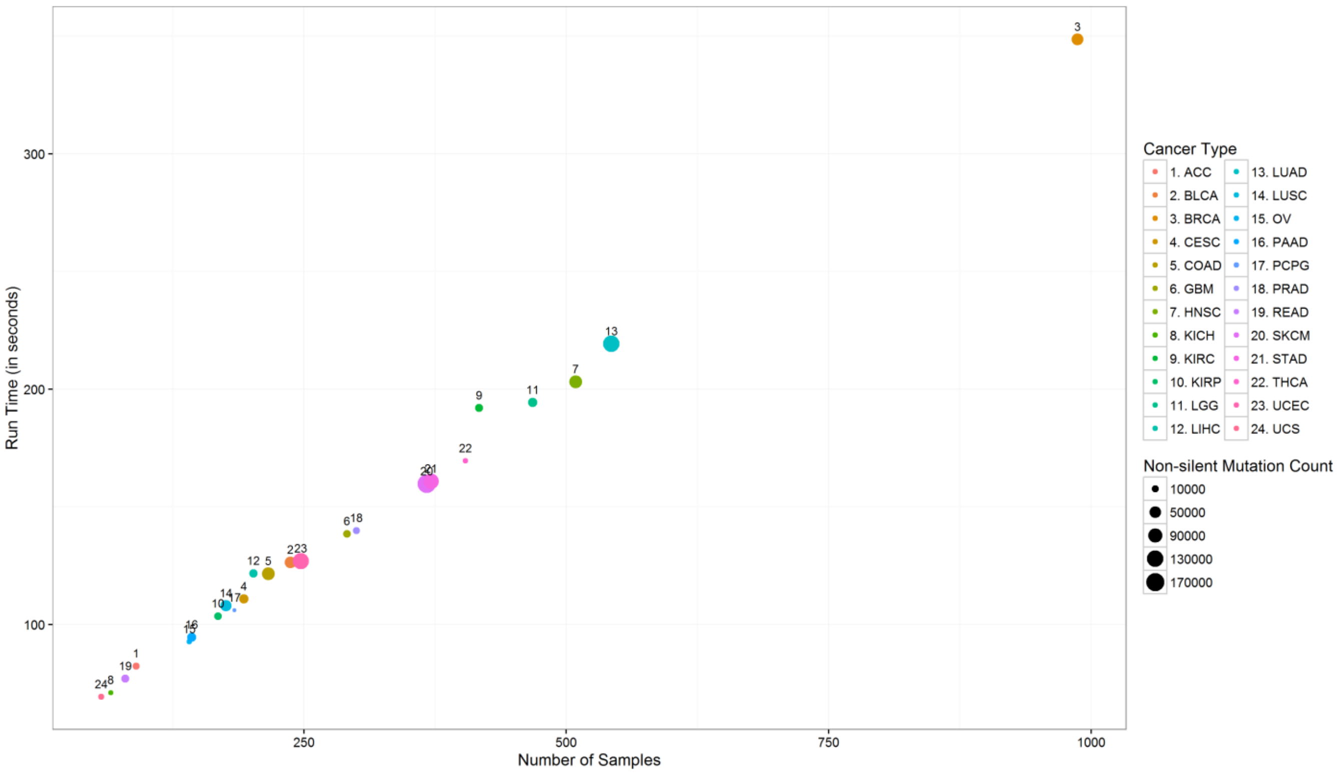

DiffMut runs significantly faster than MutSigCV [3], the method developed by Youn and Simon (YS) [4], OncodriveFML [6], and MADGiC [7]. While DiffMut is slightly slower than OncodriveCLUST [5] it significantly outperforms OncodriveCLUST across all cancer types (see Results and Fig. 3). We use BRCA as a test case because it includes the largest number of patients and thus has the longest run times. Some methods could not be run on the described desktop configuration. In those cases, which are marked with an asterisk, we ran the code on a single node within a powerful cluster where each node has up to 64GB of RAM and up to eight 2.2-2.7 GHz cores.

Method	Run time for BRCA
DiffMut	5m 47s
MutSigCV	2h 46m 47s (*)
YS	2h 55m 15s (*)
OncodriveCLUST	< 1m
OncodriveFML	1h 44m 27s (*)
MADGiC	11h 42m 39s (*)

The power of our method increases with the number of tumor samples available. For each cancer type, we randomly selected 10, 20, 30, 40, 50, 60, 70, 80, and 90 percent of all samples and measured the AUPRC as described in the paper using only those samples for our analysis. We repeated each selection ten times. We observed that, in all cases, as the number of samples we selected increases we achieve a higher fraction of the AUPRC obtained when using all samples. In some cases (e.g., COAD), the trend line levels off, implying that we are near the maximum AUPRC achievable by our method, but in other cases (e.g., LIHC), it is clear that sequencing more samples will further increase the power of our method.

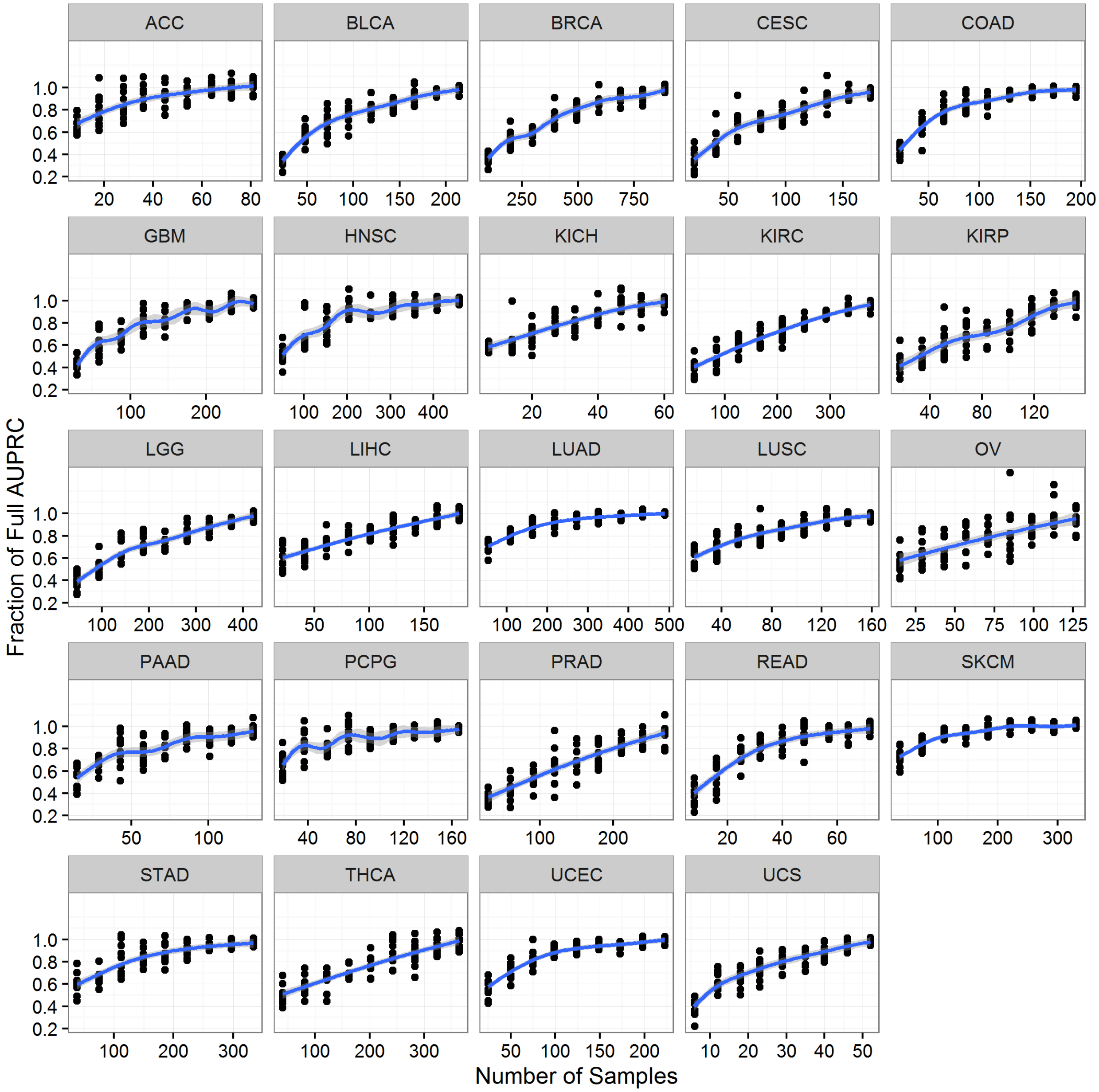

Section H: Spearman correlation with known covariates

We measured the Spearman correlation for mutated genes between their uEMD scores as computed using DiffMut with values for known covariates [3]. For reference, we also computed the Spearman correlation for mutated genes between mutation rates and these known covariates to show that our method correlates with them less and thus removes a known source of bias [3].

	**Length**		**Global Expression**		**Replication Time**		**Chromatin State**	
Type	DiffMut	Mutation Rate	DiffMut	Mutation Rate	DiffMut	Mutation Rate	DiffMut	Mutation Rate
ACC	-0.26	-0.76	0.05	-0.01	-0.08	0.04	0.09	-0.03
BLCA	0.03	-0.27	0.02	-0.06	-0.06	0.09	0.05	-0.08
BRCA	0.04	-0.27	0.01	-0.13	-0.03	0.15	0.05	-0.11
CESC	-0.10	-0.49	0.05	-0.01	-0.09	0.03	0.09	-0.02
COAD	0.03	-0.22	0.00	-0.14	-0.03	0.19	0.03	-0.17
GBM	-0.20	-0.69	0.00	-0.11	-0.01	0.17	0.02	-0.17
HNSC	0.08	-0.20	-0.07	-0.22	0.05	0.28	-0.03	-0.23
KICH	-0.32	-0.90	0.02	-0.01	-0.07	0.06	0.10	-0.03
KIRC	-0.17	-0.65	0.05	-0.02	-0.10	0.03	0.10	-0.02
KIRP	-0.23	-0.69	0.09	0.09	-0.10	-0.07	0.11	0.07
LGG	-0.02	-0.52	-0.02	-0.08	0.02	0.09	0.00	-0.09
LIHC	-0.15	-0.61	-0.01	-0.11	-0.02	0.16	0.04	-0.13
LUAD	0.11	-0.12	-0.16	-0.34	0.16	0.42	-0.12	-0.34
LUSC	-0.02	-0.36	-0.12	-0.28	0.10	0.35	-0.07	-0.29
OV	-0.34	-0.90	0.07	0.00	-0.07	0.06	0.09	-0.03
PAAD	-0.02	-0.52	-0.01	-0.05	0.01	0.09	0.01	-0.07
PCPG	-0.34	-0.82	0.05	0.10	-0.12	-0.06	0.09	0.05
PRAD	-0.26	-0.76	0.03	-0.04	-0.05	0.09	0.05	-0.07
READ	-0.18	-0.62	-0.02	-0.16	-0.02	0.18	0.03	-0.15
SKCM	0.11	-0.05	-0.11	-0.24	0.10	0.32	-0.08	-0.27
STAD	0.11	-0.07	-0.04	-0.17	0.01	0.23	0.00	-0.18
THCA	-0.34	-0.90	0.09	0.01	-0.11	0.02	0.11	-0.01
UCEC	0.08	-0.05	-0.06	-0.32	0.01	0.31	0.01	-0.26
UCS	-0.31	-0.83	0.05	-0.06	-0.07	0.07	0.08	-0.07

Section I: Olfactory receptors and extraordinarily long genes

Occurrence of olfactory receptors and extraordinarily long genes among the genes found by DiffMut (*q*-value < 0.1), MutSigCV (*q*-value < 0.1), YS (FDR < 0.1), MADGiC (FDR < 0.1), OncodriveClust (*q*-value < 0.1), and OncodriveFML (*q*-value < 0.1). The *p*-values are computed using the hypergeometric distribution to test for enrichment of these genes in the number of genes output by each method. The extraordinarily long genes consist of the ten longest genes that have above average mutation rates, with the exception of MLL2 which is in the CGC (these genes are: *TTN*, *MUC16*, *OBSCN*, *SYNE1*, *GPR98*, *AHNAK2*, *MUC5B*, *HMCN1*, and *MUC4*). There are 372 olfactory receptor genes, as annotated in HORDE [11]. Entries with a dash indicate cases where MADGiC could not be run.

	**Number of Long Mutable Genes**					
Type	DiffMut (*p*-value)	MutSigCV (*p*-value)	YS (*p*-value)	MADGiC (*p*-value)	Oncodrive CLUST (*p*-value)	Oncodrive FML (*p*-value)
ACC	0 (1.00E+00)	1 (2.79E-02)	1 (1.15E-02)	4 (7.81E-03)	0 (1.00E+00)	0 (1.00E+00)
BLCA	0 (1.00E+00)	0 (1.00E+00)	1 (8.15E-02)	- (-)	1 (1.06E-01)	0 (1.00E+00)
BRCA	0 (1.00E+00)	0 (1.00E+00)	1 (9.56E-02)	5 (5.21E-01)	0 (1.00E+00)	0 (1.00E+00)
CESC	0 (1.00E+00)	0 (1.00E+00)	1 (5.42E-02)	5 (7.54E-02)	0 (1.00E+00)	0 (1.00E+00)
COAD	0 (1.00E+00)	0 (1.00E+00)	2 (4.04E-03)	- (-)	0 (1.00E+00)	0 (1.00E+00)
GBM	0 (1.00E+00)	0 (1.00E+00)	0 (1.00E+00)	3 (5.56E-02)	0 (1.00E+00)	0 (1.00E+00)
HNSC	0 (1.00E+00)	0 (1.00E+00)	1 (1.30E-01)	- (-)	1 (1.58E-01)	0 (1.00E+00)
KICH	0 (1.00E+00)	0 (1.00E+00)	0 (1.00E+00)	1 (2.20E-02)	0 (1.00E+00)	0 (1.00E+00)
KIRC	0 (1.00E+00)	0 (1.00E+00)	1 (1.42E-02)	6 (3.57E-03)	0 (1.00E+00)	0 (1.00E+00)
KIRP	0 (1.00E+00)	0 (1.00E+00)	0 (1.00E+00)	6 (8.98E-04)	0 (1.00E+00)	0 (1.00E+00)
LGG	0 (1.00E+00)	0 (1.00E+00)	2 (3.54E-04)	5 (9.33E-02)	0 (1.00E+00)	0 (1.00E+00)
LIHC	0 (1.00E+00)	4 (3.34E-01)	1 (3.10E-02)	- (-)	0 (1.00E+00)	0 (1.00E+00)
LUAD	0 (1.00E+00)	0 (1.00E+00)	1 (1.96E-01)	7 (3.94E-01)	1 (3.08E-01)	0 (1.00E+00)
LUSC	0 (1.00E+00)	0 (1.00E+00)	2 (1.07E-03)	3 (5.76E-01)	0 (1.00E+00)	0 (1.00E+00)
OV	0 (1.00E+00)	0 (1.00E+00)	1 (3.69E-03)	0 (1.00E+00)	0 (1.00E+00)	0 (1.00E+00)
PAAD	0 (1.00E+00)	0 (1.00E+00)	0 (1.00E+00)	- (-)	0 (1.00E+00)	0 (1.00E+00)
PCPG	0 (1.00E+00)	0 (1.00E+00)	0 (1.00E+00)	- (-)	2 (1.59E-03)	0 (1.00E+00)
PRAD	0 (1.00E+00)	1 (8.75E-03)	0 (1.00E+00)	- (-)	0 (1.00E+00)	0 (1.00E+00)
READ	0 (1.00E+00)	0 (1.00E+00)	1 (1.29E-02)	- (-)	0 (1.00E+00)	0 (1.00E+00)
SKCM	0 (1.00E+00)	0 (1.00E+00)	2 (3.28E-02)	- (-)	0 (1.00E+00)	1 (3.82E-02)
STAD	0 (1.00E+00)	0 (1.00E+00)	2 (2.18E-02)	- (-)	0 (1.00E+00)	0 (1.00E+00)
THCA	0 (1.00E+00)	0 (1.00E+00)	0 (1.00E+00)	- (-)	0 (1.00E+00)	0 (1.00E+00)
UCEC	0 (1.00E+00)	0 (1.00E+00)	1 (2.00E-01)	- (-)	0 (1.00E+00)	0 (1.00E+00)
UCS	0 (1.00E+00)	0 (1.00E+00)	0 (1.00E+00)	5 (2.86E-05)	0 (1.00E+00)	0 (1.00E+00)

	Number of Olfactory Receptor Genes					
Type	DiffMut (*p*-value)	MutSigCV (*p*-value)	YS (*p*-value)	MADGiC (*p*-value)	Oncodrive CLUST (*p*-value)	Oncodrive FML (*p*-value)
ACC	0 (1.00E+00)	0 (1.00E+00)	1 (3.83E-01)	45 (8.39E-02)	0 (1.00E+00)	0 (1.00E+00)
BLCA	0 (1.00E+00)	0 (1.00E+00)	2 (8.68E-01)	- (-)	3 (8.40E-01)	0 (1.00E+00)
BRCA	0 (1.00E+00)	2 (6.30E-01)	1 (9.85E-01)	212 (9.81E-03)	9 (1.11E-02)	0 (1.00E+00)
CESC	0 (1.00E+00)	0 (1.00E+00)	0 (1.00E+00)	56 (1.00E+00)	0 (1.00E+00)	0 (1.00E+00)
COAD	0 (1.00E+00)	1 (6.55E-01)	2 (9.15E-01)	- (-)	0 (1.00E+00)	0 (1.00E+00)
GBM	0 (1.00E+00)	0 (1.00E+00)	3 (1.44E-02)	85 (5.77E-13)	0 (1.00E+00)	0 (1.00E+00)
HNSC	0 (1.00E+00)	0 (1.00E+00)	3 (9.28E-01)	- (-)	22 (2.55E-06)	0 (1.00E+00)
KICH	0 (1.00E+00)	0 (1.00E+00)	0 (1.00E+00)	0 (1.00E+00)	0 (1.00E+00)	0 (1.00E+00)
KIRC	0 (1.00E+00)	0 (1.00E+00)	0 (1.00E+00)	51 (1.00E+00)	0 (1.00E+00)	0 (1.00E+00)
KIRP	0 (1.00E+00)	0 (1.00E+00)	0 (1.00E+00)	11 (1.00E+00)	0 (1.00E+00)	0 (1.00E+00)
LGG	0 (1.00E+00)	0 (1.00E+00)	2 (3.33E-01)	109 (5.61E-01)	0 (1.00E+00)	1 (2.07E-01)
LIHC	0 (1.00E+00)	161 (1.04E-05)	1 (7.31E-01)	- (-)	0 (1.00E+00)	0 (1.00E+00)
LUAD	1 (9.80E-01)	2 (6.44E-01)	8 (6.73E-01)	294 (3.36E-07)	43 (4.46E-10)	12 (2.56E-02)
LUSC	0 (1.00E+00)	0 (1.00E+00)	4 (1.53E-01)	189 (4.06E-15)	27 (3.31E-11)	0 (1.00E+00)
OV	0 (1.00E+00)	0 (1.00E+00)	0 (1.00E+00)	0 (1.00E+00)	0 (1.00E+00)	0 (1.00E+00)
PAAD	0 (1.00E+00)	15 (8.31E-01)	0 (1.00E+00)	- (-)	0 (1.00E+00)	1 (3.46E-01)
PCPG	0 (1.00E+00)	2 (4.73E-01)	0 (1.00E+00)	- (-)	2 (7.21E-01)	0 (1.00E+00)
PRAD	0 (1.00E+00)	0 (1.00E+00)	0 (1.00E+00)	- (-)	0 (1.00E+00)	0 (1.00E+00)
READ	0 (1.00E+00)	0 (1.00E+00)	1 (4.18E-01)	- (-)	0 (1.00E+00)	0 (1.00E+00)
SKCM	0 (1.00E+00)	0 (1.00E+00)	10 (7.75E-01)	- (-)	21 (5.87E-10)	1 (8.03E-01)
STAD	0 (1.00E+00)	4 (1.28E-01)	7 (8.58E-01)	- (-)	23 (1.93E-07)	2 (9.36E-01)
THCA	0 (1.00E+00)	1 (2.51E-01)	1 (1.09E-01)	- (-)	0 (1.00E+00)	0 (1.00E+00)
UCEC	0 (1.00E+00)	0 (1.00E+00)	5 (9.52E-01)	- (-)	4 (2.80E-01)	1 (7.56E-01)
UCS	0 (1.00E+00)	0 (1.00E+00)	0 (1.00E+00)	16 (7.27E-01)	0 (1.00E+00)	0 (1.00E+00)

References

[1] TCGA Research Network, The Cancer Genome Atlas, (n.d.). http://cancergenome.nih.gov/.

[2] P.A. Futreal, L. Coin, M. Marshall, T. Down, T. Hubbard, R. Wooster, N. Rahman, M.R. Stratton, A census of human cancer genes., Nat. Rev. Cancer. 4 (2004) 177–183. doi:10.1038/nrc1299.

[3] M.S. Lawrence, P. Stojanov, P. Polak, G. V Kryukov, K. Cibulskis, A. Sivachenko, S.L. Carter, C. Stewart, C.H. Mermel, S. a Roberts, A. Kiezun, P.S. Hammerman, A. McKenna, Y. Drier, L. Zou, A.H. Ramos, T.J. Pugh, N. Stransky, E. Helman, J. Kim, C. Sougnez, L. Ambrogio, E. Nickerson, E. Shefler, M.L. Cortés, D. Auclair, G. Saksena, D. Voet, M. Noble, D. DiCara, P. Lin, L. Lichtenstein, D.I. Heiman, T. Fennell, M. Imielinski, B. Hernandez, E. Hodis, S. Baca, A.M. Dulak, J. Lohr, D.-A. Landau, C.J. Wu, J. Melendez-Zajgla, A. Hidalgo-Miranda, A. Koren, S. a McCarroll, J. Mora, R.S. Lee, B. Crompton, R. Onofrio, M. Parkin, W. Winckler, K. Ardlie, S.B. Gabriel, C.W.M. Roberts, J. a Biegel, K. Stegmaier, A.J. Bass, L. a Garraway, M. Meyerson, T.R. Golub, D. a Gordenin, S. Sunyaev, E.S. Lander, G. Getz, Mutational heterogeneity in cancer and the search for new cancer-associated genes., Nature. 499 (2013) 214–8. doi:10.1038/nature12213.

[4] A. Youn, R. Simon, Identifying cancer driver genes in tumor genome sequencing studies, Bioinformatics. 27 (2011) 175–181. doi:10.1093/bioinformatics/btq630.

[5] D. Tamborero, A. Gonzalez-Perez, N. Lopez-Bigas, OncodriveCLUST: Exploiting the positional clustering of somatic mutations to identify cancer genes, Bioinformatics. 29 (2013) 2238–2244. doi:10.1093/bioinformatics/btt395.

[6] L. Mularoni, R. Sabarinathan, J. Deu-Pons, A. Gonzalez-Perez, N. López-Bigas, OncodriveFML: a general framework to identify coding and non-coding regions with cancer driver mutations., Genome Biol. 17 (2016) 128. doi:10.1186/s13059-016-0994-0.

[7] K.D. Korthauer, C. Kendziorski, MADGiC: A model-based approach for identifying driver genes in cancer, Bioinformatics. 31 (2014) 1526–1535. doi:10.1093/bioinformatics/btu858.

[8] B. Vogelstein, N. Papadopoulos, V.E. Velculescu, S. Zhou, L.A. Diaz Jr., K.W. Kinzler, Cancer Genome Landscapes, Science (80-.). 339 (2013) 1546–1558. doi:10.1126/science.1235122.

[9] C. Kandoth, M.D. McLellan, F. Vandin, K. Ye, B. Niu, C. Lu, M. Xie, Q. Zhang, J.F. McMichael, M. a Wyczalkowski, M.D.M. Leiserson, C. a Miller, J.S. Welch, M.J. Walter, M.C. Wendl, T.J. Ley, R.K. Wilson, B.J. Raphael, L. Ding, Mutational landscape and significance across 12 major cancer types., Nature. 502 (2013) 333–9. doi:10.1038/nature12634.

[10] Y. Fu, Z. Liu, S. Lou, J. Bedford, X.J. Mu, K.Y. Yip, E. Khurana, M. Gerstein, T.P. Pdf, G. Biology, Y. Fu, Z. Liu, S. Lou, J. Bedford, X.J. Mu, E. Khurana, M. Gerstein, I. Article, A. Url, P. Central, B. Central, FunSeq2: A framework for prioritizing noncoding regulatory variants in cancer, Genome Biol. 15 (2014) 480. doi:10.1186/s13059-014-0480-5.

[11] T. Olender, N. Nativ, D. Lancet, HORDE: comprehensive resource for olfactory receptor genomics., Methods Mol. Biol. 1003 (2013) 23–38. doi:10.1007/978-1-62703-377-0_2.
